# Intrarenal Renin Angiotensin System Imbalance During Postnatal Life Is Associated With Increased Microvascular Density in the Mature Kidney

**DOI:** 10.3389/fphys.2020.01046

**Published:** 2020-09-01

**Authors:** Carolina Dalmasso, Alejandro R. Chade, Mariela Mendez, Jorge F. Giani, Gregory J. Bix, Kuey C. Chen, Analia S. Loria

**Affiliations:** ^1^Department of Pharmacology and Nutritional Sciences, University of Kentucky, Lexington, KY, United States; ^2^Department of Physiology and Biophysics, Medicine, and Radiology, University of Mississippi Medical Center, Jackson, MS, United States; ^3^Department of Internal Medicine, Hypertension and Vascular Research Division, Henry Ford Hospital, Detroit, MI, United States; ^4^Departments of Biomedical Sciences and Pathology, Cedars-Sinai Medical Center, Los Angeles, CA, United States; ^5^Clinical Neuroscience Research Center, Tulane University, New Orleans, LA, United States

**Keywords:** maternal separation, kidney, renin-angiotensin system, microvascular density, renal transcriptome

## Abstract

Environmental stress during early life is an important factor that affects the postnatal renal development. We have previously shown that male rats exposed to maternal separation (MatSep), a model of early life stress, are normotensive but display a sex-specific reduced renal function and exacerbated angiotensin II (AngII)-mediated vascular responses as adults. Since optimal AngII levels during postnatal life are required for normal maturation of the kidney, this study was designed to investigate both short- and long-term effect of MatSep on (1) the renal vascular architecture and function, (2) the intrarenal renin-angiotensin system (RAS) components status, and (3) the genome-wide expression of genes in isolated renal vasculature. Renal tissue and plasma were collected from male rats at different postnatal days (P) for intrarenal RAS components mRNA and protein expression measurements at P2, 6, 10, 14, 21, and 90 and microCT analysis at P21 and 90. Although with similar body weight and renal mass trajectories from P2 to P90, MatSep rats displayed decreased renal filtration capacity at P90, while increased microvascular density at both P21 and P90 (*p* < 0.05). MatSep increased renal expression of renin, and angiotensin type 1 (AT1) and type 2 (AT2) receptors (*p* < 0.05), but reduced ACE2 mRNA expression and activity from P2-14 compared to controls. However, intrarenal levels of AngII peptide were reduced (*p* < 0.05) possible due to the increased degradation to AngIII by aminopeptidase A. In isolated renal vasculature from neonates, Enriched Biological Pathways functional clusters (EBPfc) from genes changed by MatSep reported to modulate extracellular structure organization, inflammation, and pro-angiogenic transcription factors. Our data suggest that male neonates exposed to MatSep could display permanent changes in the renal microvascular architecture in response to intrarenal RAS imbalance in the context of the atypical upregulation of angiogenic factors.

## Introduction

Recent statistics show that nearly 40 million adults in the United States are estimated to have chronic kidney disease (CKD), while around 250 deaths per day are a consequence of end-stage renal disease (Centers for Disease Control Prevention, 2019). The majority of patients with CKD develop hypertension, a risk factor for cardiovascular disease (Ku et al., 2019). A healthy renal function is determined by genetic and environmental factors including low birth weight and intrauterine growth restriction (Franco et al., 2012; Ponzio et al., 2012; Souza et al., 2017). Particularly, both *in utero* and early life are periods of high tissue plasticity, susceptible to stressors and insults that impair the normal development of neuroendocrine, inflammatory, hormonal and autonomic responses (Zandi-Nejad et al., 2006; Hershkovitz et al., 2007; Stangenberg et al., 2015). From birth to age 4, congenital abnormalities and hereditary diseases are the leading causes of kidney disease (US Renal Data System, 2010). However, there is limited understanding of the pathophysiology by which psychosocial factors contribute to kidney disease.

Early life stress (ELS), or chronic behavioral stress during childhood, has been established as an independent cardiovascular disease risk factor (Su et al., 2015; Murphy et al., 2017). Overall, models of fetal programming of cardiovascular disease have been designed to test the effect of environmental stressors including perinatal low-protein diet, growth restriction or beta-dexamethasone exposure (Hershkovitz et al., 2007). These approaches induce a dramatic reduction in glomerular number, with a subsequent renal damage and development of hypertension. The human kidney is functional beginning at week 10 of gestation, and diuresis rate is 10 ml/hr at 32 weeks of gestation (Campbell et al., 1973); however, its maturation is completed during the next several years. In rodents, the developing kidney is particularly vulnerable to adverse perinatal environments affecting both the early and the late nephrogenic period, which will result in impaired renal excretory capacity later in life (Chen et al., 2004; Singh et al., 2013; Walton et al., 2018). Moreover, a hallmark of these models is the well-defined role of the intrarenal renin-angiotensin system (RAS) as a prerequisite for a normal nephron endowment (Guron and Friberg, 2000; Lasaitiene et al., 2003). Conversely, the pharmacological blockade of either angiotensin converting enzyme (ACE) or angiotensin II (AngII) type 1 (AT1) receptor during late nephrogenesis (postnatal days 2–14) impair renal maturation and is associated with the development of hypertension later in life (Loria et al., 2007; Saez et al., 2007).

Maternal separation (MatSep) is a chronic behavioral stress model that mimics the effects of ELS on behavioral, neuroendocrine, metabolic and cardiovascular responses (Loria et al., 2010; De Miguel et al., 2018). In previous studies, we have reported that adult male rats exposed to MatSep are normotensive and display reduced glomerular filtration rate (GFR) (Loria et al., 2013a; Loria and Osborn, 2017) and enhanced sensitivity to *in vitro* and *in vivo* AngII-mediated responses (Loria et al., 2010, 2011). On the other hand, adult female MatSep rats are normotensive but do not undergo impaired GFR or signs of proteinuria (Loria et al., 2013b). Furthermore, female MatSep rats show exacerbated AngII-induced hypertension independent of any significant worsening of the renal function compared to control littermates. In this regard, several studies have shown that hypersensitization to AngII after *in utero* exposure to low pressor doses of AngII occurs in adult male rats only (Johnson and Xue, 2018). This phenomenon can be reversed by renal denervation or ACE inhibitors (Xue et al., 2017). Thus, postnatal stress may exert the sensitization of the renal system via alteration of neuroendocrine, sympathetic and/or immune responses in a sex-specific manner.

As AngII has been shown to play a crucial role in the stimulation of vasculogenesis and angiogenesis during renal development (Sequeira Lopez and Gomez, 2004), MatSep-induced intrarenal imbalance of RAS components during postnatal life could result in permanent structural and/or functional alterations on male rats’ kidneys. Therefore, we investigated the effect of MatSep at different timepoints from neonatal to adult life, in order to determine (1) the renal vascular architecture and function, (2) the intrarenal renin-angiotensin system (RAS) components status, and (3) the genome-wide expression of genes in isolated renal vasculature, with the goal to create an integrative view of underlying mechanisms by which MatSep impact the normal renal structure and function in male rats.

## Materials and Methods

### Maternal Separation (MatSep) Protocol

All experiments were conducted per the National Institutes of Health Guide for the Care and Use of Laboratory Animals, approved and monitored by the University of Kentucky Institutional Animal Care and Use Committee. MatSep was performed using offspring from Wistar Kyoto breeders. All pups were removed from their dam’s cage from postnatal days 2 to 14 of life at the same time of day by transferring the pups to a clean cage in an incubator (30 ± 1°C) for 3 h. The control group was the non-handled litters that remained with their mother (Loria et al., 2010; De Miguel et al., 2017). Different samples were taken under light isoflurane anesthesia at postnatal day 2 (P2), P6, P10, P14, P21, P90, and P180. Female littermates were kept for sampling or included in other studies.

### Experimental Design

Groups were comprised of male rats from at least 4 different litters. Littermates were randomized at different time points. In a first subset, body weight, and kidney weights were recorded while plasma and renal tissue were collected at different timepoints and kidney gene and protein expression in frozen tissue were determined at different timepoints. The whole kidney for P2–P21 and renal cortex for P90 time points were used in the experiments. In this subset, the glomerular filtration rate (GFR) was determined at P21 and P90 in randomized littermates. In a second subset, kidneys were collected in neonates (P10) and adults (P180) to isolate the renal vasculature and perform a genome-wide transcriptome assays using Affymetrix GeneChip microarrays. In the third subset of offspring, male rats were perfused at P21 and P90 with a silicone polymer, and kidneys were collected to measure the microvascular density.

### Micro-Computerized Tomography

A saline-filled cannula was placed in the aorta, the aorta was ligated below and above the renal arteries, and infusion of 0.9% saline (containing 10 units/mL heparin) was initiated under physiological perfusion pressure at a rate of 2 mL/min (Syringe Infusion Pump 22; Harvard Apparatus, Holliston, MA, United States). A small incision was performed in the inferior vena cava to allow the saline infusion to drain. After 10–15 min of saline infusion and when it drained freely from the vein, it was immediately replaced with an infusion of intravascular contrast agent (2 mL/min), which was a freshly mixed radio-opaque silicone polymer contrast containing lead chromate (SkyScan 1076, Bruker Biospin Corp., MA, United States) until the polymer drained freely from the vein as previously reported (Flynn et al., 2013; Tullos et al., 2015). Then, the polymer-filled kidneys were left at 4°C overnight and then immersed in 10% buffered formalin for 72 h before scanning. The kidney samples were scanned at 0.3° increments using a micro-CT scanner (SkyScan 1076 system; Micro Photonics, Inc., Allentown, PA, United States), and the X-ray transmission images were acquired in each angle of view at a resolution of 18 μm and digitized to 16 bits grayscale. Three-dimensional (3D) volume images were reconstructed using a filtered back-projection algorithm and displayed on a computer workstation by volume rendering for display and analysis of renal MV using the Analyze software package (Biomedical Imaging Resource; Mayo Clinic, Rochester, MN, United States).

### Western Blot

The whole kidney at P21 was homogenized and protein concentration was determined using the Bradford assay (Bio-Rad), and then samples containing 30 μg protein/well were loaded in 10% SDS-PAGE and transferred onto PVDF membranes. Membranes were incubating in blocking buffer (5% non-fat dry milk/TBS) for 1 h at room temperature, followed by overnight incubation at 4°C in the presence of antibodies directed against Perlecan primary antibody 1:250 (#sc-25848, Santa Cruz, CA, United States), and GAPDH primary antibody 1:10,000 (#GTX100118, GeneTex). Membranes were washed with TBS–0.1% Tween 20 and incubated in the presence of HRP-conjugated secondary antibody 1:20,000 (#926-32211, 926-68020). Band detection was performed by the LiCor Odyssey imaging system. Blot quantification was performed using NIH ImageJ software.

### Immunohistochemistry

Whole kidneys were collected at P21 and frozen in OCT. Tissue was cut in 20 μm sections using a cryostat and directly mounted onto slides. Sections were fixed with ice-cold acetone/methanol (50:50 mixture) prior to incubating in blocking buffer (5% BSA in 1xPBS with 0.1% Triton X-100) for 1 h at room temperature. The sections were then incubated overnight at 4°C with FITC-conjugated tomato-lectin (1:200; Vector Laboratories, Burlingame, CA, United States). Slides were then washed and coverslipped with fluorescent mounting media (Vector Laboratories, Burlingame, CA, United States) and images were captured using Eclipse Ti microscope/DS-Ri1 CCD color camera and NIS analysis software (Nikon Instruments). Five images per animal were then analyzed for the number of stain-specific positive pixels using Adobe Photoshop (Adobe Inc.). Briefly, images were converted to grayscale, adjusted to a set threshold equal to the antibody staining and the number of pixels calculated. The data were averaged per group and are presented a number of positive pixels.

### Transcutaneous Glomerular Filtration Rate (tGFR)

Renal function was evaluated by transcutaneous measurement of the elimination kinetics of fluorescein isothiocyanate (FITC)-sinistrin (Mannheim Pharma & Diagnostics). Rats were placed under light anesthesia (isoflurane) and the flank area was depilated to apply the transcutaneous receiver on top (NIC-kidney device). The receiver was secured around the body with 3M surgical tape. After the animal awakened from anesthesia, a 5-min baseline trace was recorded. Then, they were injected with 30 μl of FITC-sinistrin (5 mg/100 g BW in 0.9% saline, retroorbital, Fresenius Kabi, Linz, Austria) under light isoflurane using microneedles. After 90 min of measurement, the device was removed. The probe was read to determine t_1__/__2_ in minutes. Renal function was evaluated by the elimination kinetics (three-compartmental model) using the following formula = 21.33/(t_1__/__2_) = GFR (μl/min/100 g BW).

### Intrarenal RAS Components Trajectory

#### Renal Gene Expression by RT-qPCR

RNA was extracted from kidneys using RNeasy mini kit (Qiagen, CA). Briefly, total mRNA was extracted from tissues using TRIZOL reagent (Invitrogen Life Technologies, Carlsbad, CA, United States) according to the manufacturer’s protocol. Rat forward (sense) and reverse (antisense) QuantiTect primers for GAPDH (Entrez ID: 35728), AGT (Entrez ID: 24179), renin (Entrez ID: 24715), AT_1_ receptor (Entrez ID: 24182), AT_2_ receptor (Entrez ID: 24180), ACE (Entrez ID: 24310), ACE2 (Entrez ID: 302668), MAS-1 (Entrez ID: 24180), Neprilysin (Entrez ID: 24590) and aminopeptidase A (Entrez ID: 64017) were analyzed by quantitative real-time RT-PCR as previously reported (Loria et al., 2011). GAPDH was used as a housekeeper gene. Ct values from each sample were normalized to GAPDH expression within the sample (ΔCt) followed by normalization to ΔCt values for AT2 control samples at P2 (ΔΔCt) prior to calculation of relative gene expression (2-ΔΔCt) as previously described (Loria et al., 2011).

#### Renal AGT Protein Content

Renal tissue was homogenized in ELISA buffer (∼100 mg tissue/500 μl) and diluted 1:200 following the manufacturer’s protocol (Immuno-Biological Laboratories America, Minneapolis, MN, United States) (Dalmasso et al., 2019).

#### Renal AngII Peptide Content

Kidneys were excised, drained, weighed, and homogenized in chilled inhibitor cocktail (EDTA 200 mM, PMSF 1 mM, 1,10 phenanthroline 125 mM, Pepstatin A 2 mM, Enalapril 1 mM) and 1 ml of methanol, centrifuged at 4°C for 10 min. The supernatants were dried overnight in a vacuum centrifuge (Savant, Hicksville, NY, United States). The dried residue was kept at −20°C until AngII peptide was determined by radioimmunoassays as described previously (Mitchell et al., 1997).

### Plasma Renin Concentration (PRC)

Whole blood was collected in pre-chilled EDTA-coated tubes, centrifuged at 4°C and rapidly snap-frozen in liquid nitrogen. Immediately after thawing the samples, a protease inhibitor (PMSF) was added to the sample to prevent angiotensin I (AngI) cleavage by other proteases. PRC was determined as the amount of AngI synthesized after incubation with excess of angiotensinogen as previously described (Peng et al., 2001; Mendez et al., 2011). The assay relies on the fact that as long as the concentration of substrate is not limiting, the production of AngI by renin is constant. Substrate consumption is never greater than 3% of total AngI, and therefore assures linearity over time. Thus, plasma samples were incubated with excess rat angiotensinogen at 37°C for 1.5 h, boiled for 10 min, followed by centrifugation at 16,100 *g*. Supernatants were collected and generated AngI was measured using an ELISA kit (Immuno-Biological Laboratories, Minneapolis, MN, United States). Values were expressed as ng AngI/ml, generated per hour of incubation.

### Renal Vasculature Isolation

Following euthanasia, kidneys from neonates (P10) or adults (P180) were removed and immediately placed into a petri dish with cold physiological saline solution (PSS). After removal of the renal capsule, the kidney was placed between a circle sieve of 70-μm pore size for neonates and 100-μm pore size for adult rats (Biodesign, Carmel, NY, United States) as previously reported (Loria and Osborn, 2017). Kidney vessels were immediately isolated by rapid and gentle grating. The kidney vessels were subsequently frozen in liquid nitrogen and stored at −80°C.

### ACE and ACE2 Activity in Neonatal Kidneys

Both ACE and ACE2 activities were measured using fluorescence assays, as previously described (Eriguchi et al., 2018). Briefly, whole snap-frozen kidneys were gently homogenized in 20 mM HEPES (pH 7.3) and centrifuged at 3,000 *g* for 15 min at 4°C. The supernatant was discarded, and the pellets were vigorously re-homogenized in 20 mM HEPES with 0.5% Triton X-100 (pH 7.3). After a second centrifugation at 20,000 *g* for 20 min at 4°C, the supernatant was collected and the protein concentration was determined using a Pierce BCA protein assay kit (Thermo Scientific, Rockford, IL, United States). The ACE activity was measured using 2 μg of protein extract and 10 μM of the fluorescent substrate (Mca-Arg-Pro-Pro-Gly-Phe-Ser-Ala-Phe-Lys(Dnp)-OH (R&D Systems, Minneapolis, MN, United States) with and without the 2 μM ACE inhibitor captopril. ACE2 activity was measured using 3 μg of protein extract and 50 μM of the fluorescent substrate Mca-Ala-Pro-Lys(Dnp)-OH (Anaspec, Fremont, CA, United States) with and without 1 μM of the ACE2 inhibitor DX600. The degradation of the fluorogenic peptides (fluorescence) was measured over time in a spectrophotometer (FLUOstar Omega, BMG LABTECH) at 320 nm excitation and 405 nm emissions. Only the hydrolytic activity inhibited by the specific inhibitors was considered for calculations.

### Microarray

Frozen renal tissue was used to extract and assess RNA purity and integrity. Tissue RNA was isolated from neonates and adults MatSep rats and their control littermates (P10 and P180, respectively; *n* = 5 per group). The genome-wide analysis was performed using rat GeneChip expression RaGene2.0_st arrays (Affymetrix, Thermo Fisher Scientific, United States). The microarray assays were run in the Microarray Core Facility at the University of Kentucky. Briefly, tissue RNA was labeled using WT-IVT whole transcriptome amplification procedure (following Affymetrix rat array protocol). For each sample, the labeled probes were applied on a rat Gene2.0 ST array for hybridization overnight, followed by array scanning to obtain probe signal intensity data file. The array data files of tissue samples were further processed to obtain signal intensity for each gene transcript. Enriched Biological Pathways functional clusters (EBPfc) affected by MatSep were analyzed by gene over-representation analysis using DAVID Functional Annotation Bioinformatics Microarray Analysis v6.8^1^. Data are available at https://www.ncbi.nlm.nih.gov/geo/query/acc.cgi?acc=GSE151402. Extended microarray findings including a general validation can be found in the Supplementary Material.

### Statistical Analysis

Analysis was performed using the GraphPad Prism version 7.00 (Macintosh, GraphPad Software, La Jolla, CA, United States^2^). Data are reported as means ± SE. The criterion for significance was *p* < 0.05. Differences between control and MatSep groups between more than two-timepoints (P2-P90) were determined by two-way ANOVA. Differences in means among groups for non-repeated variables were compared by *t*-test when normality was verified.

For the statistical analysis of the microarray data, gene specific analysis (GSA) model implemented in Partek Genomics Suite (Partek, Inc., MO) was used to assess differential expression among the four experimental groups. Each RNA transcript signal intensity was first normalized to its mean value across all samples from the four experimental groups. Differentially expressed genes affected by MatSep in neonates and adult rats were identified by *post hoc* pair-wise comparisons.

## Results

### Effect of MatSep on Renal Mass and Function

**FIGURE 1:**
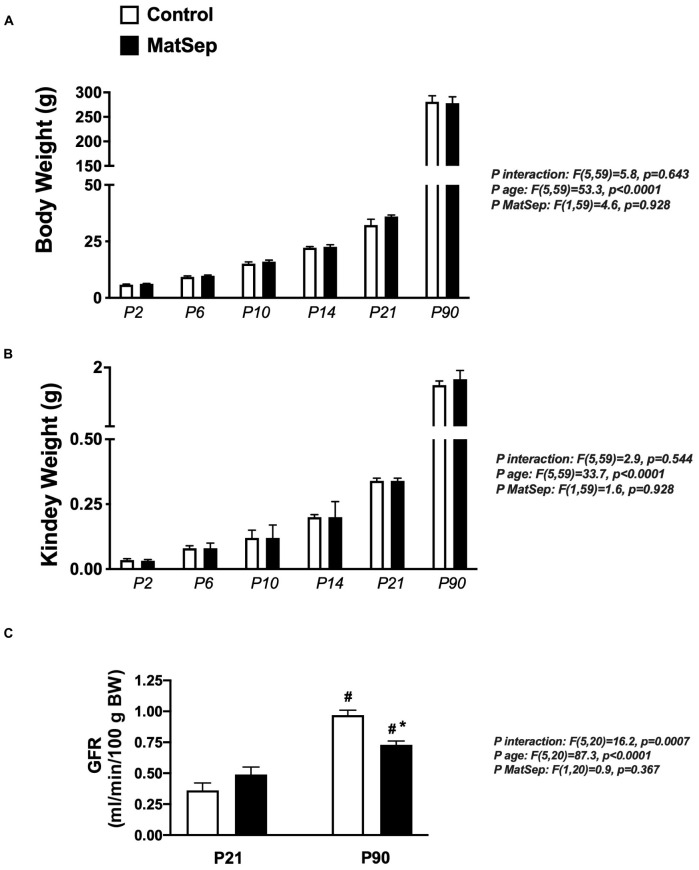
Effect of MatSep on the trajectory from neonatal to adult male rats in: **(A)** body weight, **(B)** Kidney, and **(C)** conscious GFR. #*p* < 0.05 vs. P21, *p < 0.05 vs. C. P: postnatal day. *n* = 6–8 per group.

Exposure to maternal separation did not affect the body weight trajectory from P2 to P90 in male rats (Figure 1A). In addition, MatSep and control rats displayed similar kidney weights at the same time points (Figure 1B). MatSep and control weanlings showed similar GFR. However, although older animals showed increased renal filtration capacity was increased in older rats, MatSep reduced GFR when compared with control littermates. These data indicate that MatSep induces long-term changes in renal function that are not caused by low birth weight or reduced renal mass (Figure 1C).

### Effect of MatSep on Renal Microvascular Architecture

**FIGURE 2:**
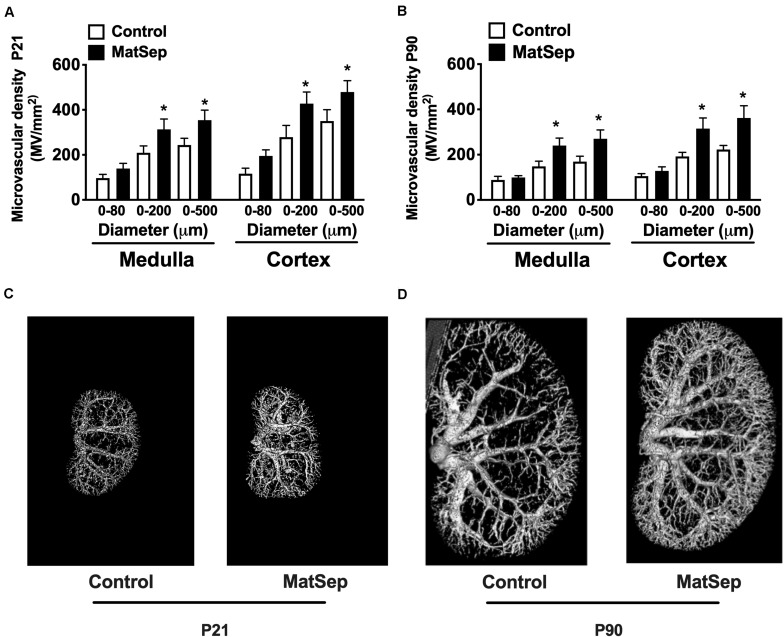
Effect of maternal separation (MatSep) on microvascular density in renal cortex and medulla at **(A)** P21 and **(B)** P90. Renal microphotographs in Control (right) and MatSep (left) at **(C)** P21 and **(D)** P90. P: postnatal day. *p < 0.05 vs. C. *n* = 5–7 per group.

In response to MatSep, the number of vessels in the 0–200 and 0–500 μM range in kidneys from 21-day-old weanlings was significantly increased in both medulla and cortex areas (Figure 2A). Similarly, microvascular density was increased in kidneys from MatSep rats at P90 (Figure 2B), suggesting that MatSep exerts early, long-lasting effects on the density of the renal microvasculature. Figures 2C,D show representative images of the renal vascular tree at P21 and P90, respectively.

**FIGURE 3:**
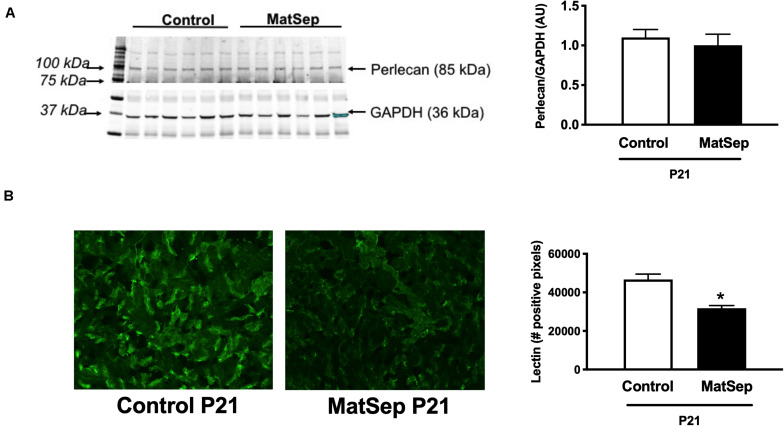
Effect of maternal separation (MatSep) on: **(A)** renal protein expression of perlecan, and **(B)** immunostaining intensity for lectin in 3-week old male weanlings (P21). *p < 0.05 vs. C. *n* = 5 per group.

Furthermore, we determined perlecan (a marker of basement membrane) and tomato-Lectin (a marker of vascular endothelium) in kidneys from P21 rats. While perlecan protein expression was similar between groups (1.1 ± 0.1 vs. 0.9 ± 0.2 AU, Figure 3A), tomato-Lectin staining was reduced in kidneys from MatSep compared to control (32687 ± 4185 vs. 52578 ± 5988 positive pixels, *p* < 0.05, Figure 3B). Thus, MatSep induces a mismatch between increased microvasculature and cells expressing vascular endothelium in kidneys.

### Effect of MatSep on Intrarenal RAS Trajectory

**FIGURE 4:**
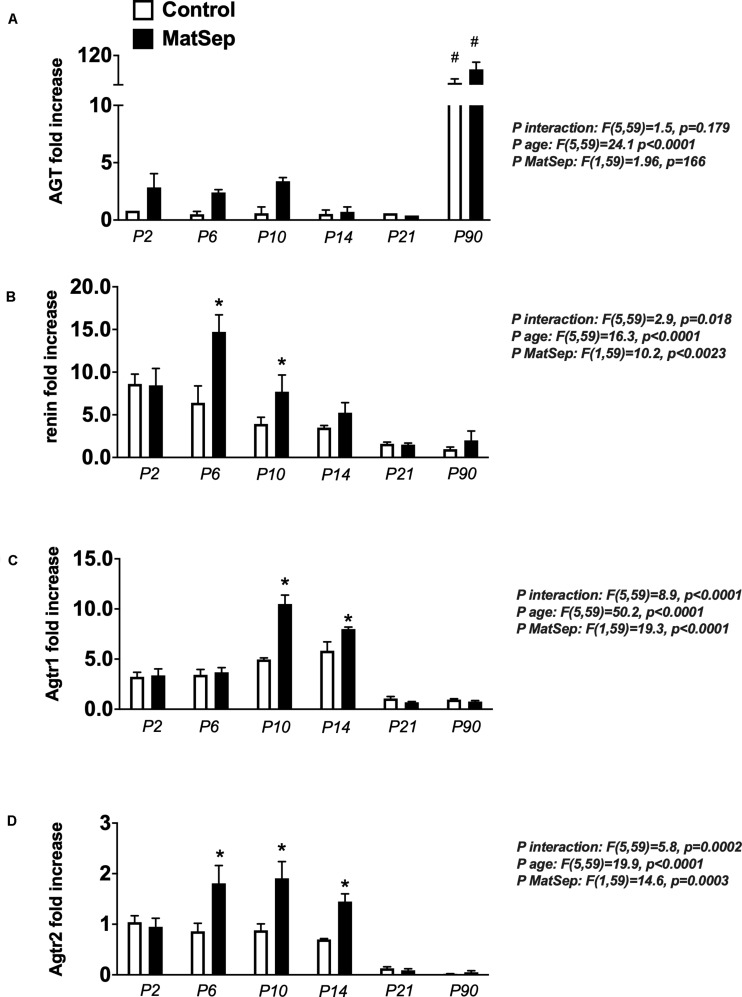
Effect of maternal separation (MatSep) on intrarenal RAS mRNA expression trajectory from P2 to P90: **(A)** Angiotensinogen (Agt), **(B)** renin, **(C)** angiotensin type 1 receptor (Agtr1) and **(D)** angiotensin type 1 receptor (Agtr2). Each RAS component was normalized to Agtr2 receptor control levels at P2. #*p* < 0.05 vs. P2–P21, *p < 0.05 vs. C. *n* = 6–8 per group.

In the neonatal kidney, angiotensinogen (AGT) mRNA expression was not statistically different among the groups (Figure 4A). Renin (Figure 4B), Agtr1 (AT1) receptors (Figure 4C) and Agtr2 (AT2) receptors (Figure 4D) mRNA expression were higher in MatSep offspring between P6-P14 compared to controls. Renin and AngII receptors mRNA abundance were similar in kidneys from MatSep and control adult rats and significantly reduced compared to neonatal levels.

**FIGURE 5:**
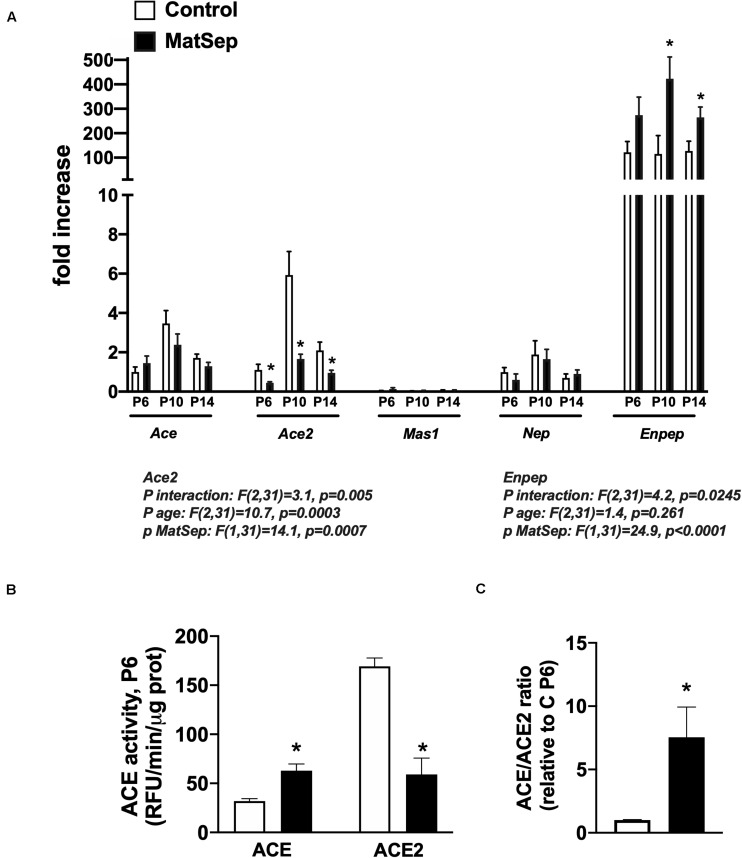
Effect of maternal separation (MatSep) on intrarenal RAS components at postnatal day 6, 10, and 14 in male neonates: **(A)** Ace, Ace2, Mas1, Nep, and Enpep; **(B)** ACE and ACE2 enzymatic activity; **(C)** ACE/ACE2 ratio. *p < 0.05 vs. C. *n* = 4–8 per group.

Notably, ACE mRNA expression was unchanged from P6-14 in MatSep neonates (Figure 5A), while ACE activity was increased (Figure 5B). However, both ACE2 mRNA and enzymatic activity were significantly reduced in kidneys from MatSep neonates compared to controls. In addition, the ACE/ACE2 ratio at P6 was ∼10 fold increased in MatSep tissue (Figure 5C). Furthermore, enpep (aminopeptidase A), another RAS enzyme which converts AngII to AngIII, was increased by MatSep during this postnatal window.

**FIGURE 6:**
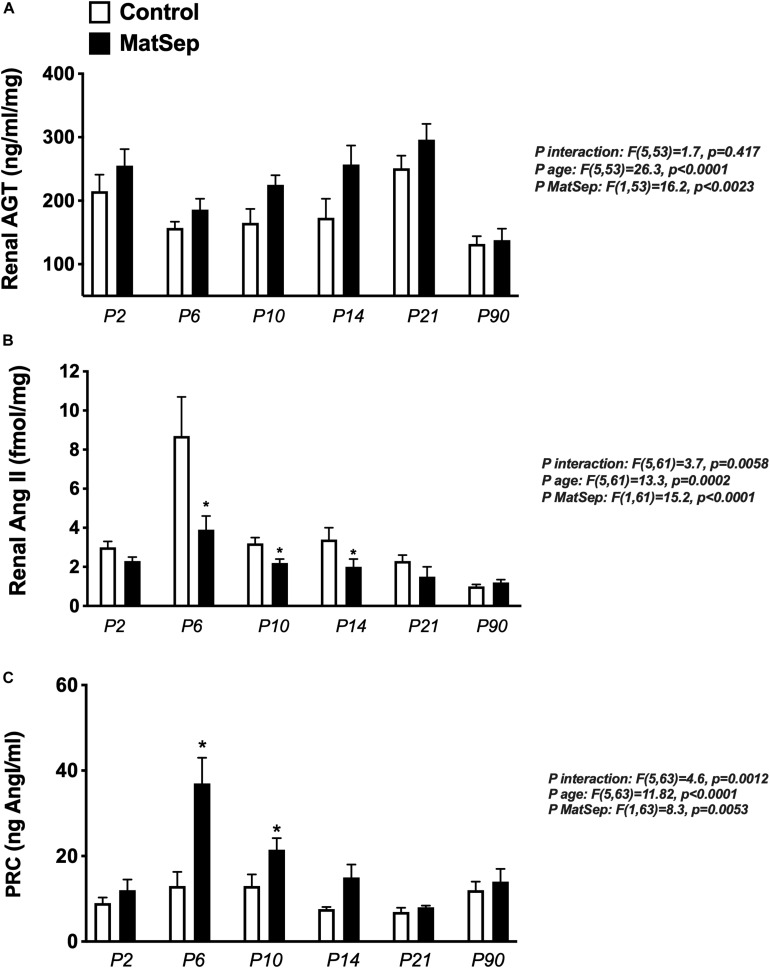
Effect of maternal separation (MatSep) on RAS trajectory from neonatal to adult life in: **(A)** renal angiotensinogen (AGT), **(B)** renal angiotensin II (AngII), and **(C)** plasma renin concentration (PRC) in ng AngI/ml generated per hour of incubation. *p < 0.05 vs. C. *n* = 6–8 per group.

Renal AGT protein content was not significantly different between groups at any age (Figure 6A); however, AngII peptide levels were reduced by MatSep at P2, 6, 10, and 14 (Figure 6B). Nevertheless, PRC was increased at P6, P10, and P14 (Figure 6C).

### Age Effect on Gene Expression in Isolated Renal Vasculature From Control Rats

Comparisons were made taking as a reference the changes in gene expression underwent by control samples from neonatal to adult life (P10 and P180). Of a total of 20258 annotated genes RNA transcripts, 12821 genes did not have significant changes in gene expression associated with age (*p* < 0.03 at step-up FDR < 0.05). However, 3745 genes showed a higher expression in neonates compared to adults and called “early life” genes. The 3692 remaining genes showed a higher expression in adults compared to neonates and were classified as “adult life” genes. A total of 489 genes altered by MatSep (FDR < 0.05). In addition, we identified 368 genes in which the expression was affected by the *MatSep x Age* interaction (FDR < 0.4). A detailed description of *MatSep x Age* interactions on isolated renal vasculature gene expression can be found in the Supplementary Excel Spreadsheet.

### Effect of MatSep on Isolated Renal Vasculature Transcriptomics

Renal vasculature of MatSep neonates showed increased RAS of (Table 1), angiogenic (Table 2) and inflammatory (Table 3) gene expression compared with controls. Ace2 transcript was not included in the rat microarray that was used for this study.

**TABLE 1:** Effect of MatSep on RAS gene expression trajectory from neonate-to-adult life.

**RefSeq**	**Gene**	**Fold Increase Neonate**	***p*-value**	**Fold Increase adult**	***p*-value**
	***Classical***				
**NM_030985**	**Agtr1a**	**1.14**	**0.020**	−1.080	0.150
**NM_012576**	**Nr3C1**	**1.11**	**0.004**	1.023	0.482
NM_134432	AGT	−1.07	0.484	−1.135	0.214
NM_012642	REN	−1.02	0.800	1.042	0.657
NM_012544	ACE	1.16	0.239	1.100	0.458
ENSRNOT00000052018	Nr3C2	−1.09	0.169	1.039	0.513
NM_017080	HSD11b1	−1.02	0.717	1.008	0.902
ENSRNOT00000023130	HSD11b2	−1.03	0.738	1.075	0.482
	***Non-classical***				
**NM_012494**	**AGTR2**	**1.59**	**0.003**	−1.082	0.563
**ENSRNOT00000021840**	**IGF2R**	**1.16**	**0.027**	1.039	0.531
NM_012757	MAS1	1.08	0.607	1.214	0.229
NM_001007091	ATP6AP2	−1.06	0.519	1.008	0.925
NM_031012	ANPEP	−1.07	0.253	−1.054	0.398
ENSRNOT00000009198	RNPEP	1.04	0.521	−1.002	0.970
	***Related***				
**ENSRNOT00000014970**	**CPA3**	**1.61**	**0.005**	−1.01	0.939
**NM_031523**	**KLK1**	**1.34**	**0.014**	1.02	0.843
NM_012608	MME	−1.22	0.088	1.04	0.702
ENSRNOT00000010831	CYP11A1	−1.08	0.197	1.04	0.724
NM_012538	CYP11b2	−1.01	0.971	1.07	0.657
NM_012537	CYP11b1	1.21	0.151	−1.22	0.136
NM_012753	CYP17A1	1.13	0.316	1.10	0.106
ENSRNOT00000006087	EGFR	1.07	0.233	−1.07	0.265
NM_001113403	LNPEP	1.02	0.661	1.00	0.926
NM_053748	DPP3	1.01	0.724	1.03	0.329
NM_134334	CTSD	−1.01	0.823	1.08	0.408
NM_001011959	CTSA	−1.02	0.838	1.05	0.634
ENSRNOT00000000360	PREP	1.00	0.951	1.01	0.902

*Fold change was calculated from control samples at P10. In bold: genes with significant increases in fold vs. control, *n* = 5 per group. Noting, ACE2 transcript was not available in the array.*

**TABLE 2:** Effect of MatSep on angiogenic gene expression trajectory from neonate-to-adult life.

**RefSeq**	**Gene**	**Fold Increase neonate**	***p*-value**	**Fold Increase adult**	***p*-value**
	***Up***				
**NM_001108644**	**Mfap5**	**2.639**	**0.0001**	−1.145	0.484
**NM_012494**	**Agtr2**	**1.589**	**0.003**	−1.082	0.563
**ENSRNOT00000015395**	**Rspo3**	**1.545**	**0.003**	−1.117	0.377
**NM_001107100**	**Col8a1**	**1.536**	**0.017**	−1.153	0.393
**NM_031970**	**Hspb1**	**1.480**	**0.012**	−1.163	0.293
**NM_030868**	**Nov**	**1.430**	**0.009**	−1.232	0.105
**ENSRNOT00000013093**	**Il18**	**1.402**	**0.006**	−1.071	0.532
**NM_001109383**	**Angptl1**	**1.309**	**0.034**	1.015	0.896
**NM_199115**	**Angptl4**	**1.281**	**0.035**	−1.247	0.057
**ENSRNOT00000038994**	**Cybb**	**1.302**	**0.002**	−1.033	0.649
**ENSRNOT00000016485**	**Fgf10**	**1.273**	**0.006**	1.170	0.055
**NM_031054**	**Mmp2**	**1.262**	**0.020**	−1.152	0.135
**NM_017154**	**Xdh**	**1.250**	**0.004**	−1.088	0.215
**ENSRNOT00000012216**	**C3ar1**	**1.241**	**0.001**	−1.099	0.101
**NM_053560**	**Chi3l1**	**1.236**	**0.004**	−1.058	0.385
**NM_012769**	**Gucy1b3**	**1.233**	**0.017**	−1.106	0.218
**NM_019185**	**Gata6**	**1.230**	**0.014**	−1.110	0.185
**NM_001107159**	**Mmp19**	**1.214**	**0.041**	−1.069	0.453
**NM_133569**	**Angptl2**	**1.203**	**0.029**	−1.062	0.450
**NM_001109093**	**Grb10**	**1.185**	**0.005**	1.006	0.913
**NM_017364**	**Zfp260**	**1.175**	**0.002**	−1.061	0.181
**NM_001106579**	**Sema3e**	**1.161**	**0.011**	1.078	0.169
**NM_134452**	**Col5a1**	**1.152**	**0.005**	−1.010	0.827
**NM_031798**	**Slc12a2**	**1.135**	**0.017**	1.053	0.290
NM_134454	Angpt2	1.026	0.674	−1.070	0.274
NM_031530	Ccl2	1.203	0.074	−1.511	0.914
ENSRNOT00000003313	Tgfb2	1.179	0.236	1.017	0.900
NM_012802	Pdgfra	1.164	0.108	−1.418	0.001
NM_053394	Klf5	1.118	0.222	1.014	0.878
NM_031525	Pdgfrb	1.080	0.474	−1.211	0.087
XM_002725723	Adam12	1.064	0.413	−1.292	0.003
NM_024400	Adamts1	1.047	0.475	1.007	0.916
ENSRNOT00000001248	Flt1	1.041	0.580	1.032	0.664
NM_053549	Vegfb	1.089	0.310	−1.054	0.525
	***Down***				
**ENSRNOT00000019210**	**Col24a1**	−**1.170**	**0.025**	−1.025	0.697
**NM_133386**	**Sphk1**	−**1.249**	**0.0002**	1.037	0.440
**NM_012801**	**Pdgfa**	−**1.247**	**0.001**	−1.006	0.908
**NR_031903**	**Mir185**	−**1.231**	**0.002**	−1.005	0.925
NM_133286	Fgf8	−1.042	0.416	−1.084	0.122
ENSRNOT00000009669	Amot	−1.080	0.097	1.027	0.550

*Fold change was calculated from control samples at P10. In bold: genes with significant increases vs. C.*

**TABLE 3:** Effect of MatSep on inflammatory gene expression trajectory from neonate-to-adult life.

**RefSeq**	**Gene**	**Fold Increase neonate**	***p*-value**	**Fold Increase adult**	***p*-value**
	***Up***				
**ENSRNOT00000014701**	**Fabp4**	**3.737**	**0.015**	−1.321	0.571
**NM_001008513**	**Ccl21**	**1.695**	**0.003**	−1.38	0.051
**NM_001145366**	**Pparg**	**1.585**	**0.040**	−1.015	0.942
**ENSRNOT00000000172**	**Cdo1**	**1.540**	**0.007**	1.086	0.565
**NM_031970**	**Hspb1**	**1.480**	**0.012**	−1.163	0.293
**NM_013174**	**Tgfb3**	**1.465**	**0.003**	−1.182	0.150
**NM_031504**	**C4a**	**1.464**	**0.002**	−1.123	0.589
**NM_030868**	**Nov**	**1.430**	**0.009**	−1.232	0.105
**ENSRNOT00000021064**	**Abcd2**	**1.412**	**0.001**	−1.13	0.181
**ENSRNOT00000013093**	**Il18**	**1.402**	**0.006**	−1.071	0.532
**NM_012705**	**Cd4**	**1.401**	**0.002**	−1.038	0.681
**ENSRNOT00000037681**	**Nfam1**	**1.384**	**0.0003**	−1.087	0.262
**NM_053611**	**Nupr1**	**1.384**	**0.005**	−1.081	0.447
**ENSRNOT00000020265**	**Ifitm3**	**1.375**	**0.009**	−1.043	0.703
**NM_001013427**	**Rarres2**	**1.359**	**0.002**	−1.146	0.109
**NM_012870**	**Tnfrsf11b**	**1.353**	**0.015**	1.045	0.699
**NM_001131001**	**Fcer1g**	**1.328**	**0.003**	−1.020	0.809
**ENSRNOT00000024110**	**Ccdc3**	**1.313**	**0.002**	−1.019	0.802
**ENSRNOT00000028131**	**Axl**	**1.300**	**0.009**	−1.095	0.324
**NM_001134545**	**Ssc5d**	**1.291**	**0.0004**	−1.090	0.151
**ENSRNOT00000005686**	**Irak3**	**1.288**	**0.003**	−1.035	0.639
**ENSRNOT00000021397**	**Dpep1**	**1.287**	**0.012**	−1.423	0.001
**ENSRNOT00000009993**	**Casp1**	**1.286**	**0.009**	−1.025	0.247
**ENSRNOT00000018328**	**Tgm2**	**1.272**	**0.002**	−1.163	0.035
**ENSRNOT00000045867**	**Ccl6**	**1.257**	**0.014**	1.013	0.880
**NM_053560**	**Chi3l1**	**1.236**	**0.004**	−1.058	0.385
**NM_013110**	**Il7**	**1.209**	**0.013**	−1.017	0.813
**NM_001008722**	**Irf8**	**1.201**	**0.004**	−1.076	0.195
**NM_173045**	**Ripk2**	**1.196**	**0.002**	−1.062	0.242
**NM_001106418**	**Il7r**	**1.189**	**0.016**	−1.136	0.067
**NM_001257278**	**Il31ra**	**1.178**	**0.017**	−1.284	0.001
**NM_138879**	**Sele**	**1.173**	**0.046**	−1.034	0.659
**NM_013185**	**Hck**	**1.172**	**0.015**	1.053	0.391
**ENSRNOT00000020108**	**Il1rl1**	**1.171**	**0.049**	−1.149	0.083
**ENSRNOT00000025222**	**Csf1**	**1.170**	**0.006**	−1.059	0.703
**ENSRNOT00000014560**	**Birc2**	**1.150**	**0.002**	1.027	0.474
**ENSRNOT00000019673**	**Il1r1**	**1.138**	**0.014**	−1.196	0.001
**NM_145789**	**Il13ra1**	**1.117**	**0.015**	−1.023	0.582
**NM_138502**	**Mgll**	**1.115**	**0.016**	1.032	0.443
**ENSRNOT00000004285**	**Gne**	**1.085**	**0.044**	−1.045	0.350
NM_020542	Ccr1	1.578	0.0001	−1.262	0.014
XM_001058423	Eda	1.135	0.095	−1.245	0.007
XM_001056441	C2cd4b	1.071	0.231	−1.032	0.569
ENSRNOT00000002089	Cd80	1.054	0.249	−1.326	0.0001
NM_001109112	Tnfsf13b	1.060	0.391	−1.292	0.001
NM_012675	Tnf	1.067	0.241	−1.143	0.023
NM_001013894	Lilrb4	−1.083	0.262	−1.289	0.002
	***Down***				
**ENSRNOT00000000621**	**Mapk13**	−**1.272**	0.004	−1.021	0.779
**NM_133386**	**Sphk1**	−**1.249**	0.0002	1.037	0.440
NM_012854	Il10	−1.044	0.399	−1.120	0.036
NM_031512	Il1b	−1.066	0.402	−1.197	0.028

*Fold change was calculated from control samples at P10. In bold: genes with significant increases in fold vs. control, *n* = 5 per group.*

Furthermore, MatSep induced downstream effects in a group of genes, whereas some of them were also linked to inflammation and angiogenesis (Supplementary Table S2). These genes were expressed similarly between groups in neonates but significantly changed by MatSep in adult rats. The 25 upregulated genes participate in diverse metabolic processes, embryonic cranium morphogenesis, anatomical structure development, circadian regulation of gene expression as well as ion homeostasis.

Table 4 shows that EBPfc affected by MatSep in neonates and adult rats are mostly related to cell proliferation and immune system activation. Finally, due to the fact that MatSep changes the vascular architecture of the kidney, we validated the tubular contamination and the proportion of vascular vs. non-vascular cells by RT-qPCR in our preparation. Similar to lectin, the vascular endothelium marker PECAM-1 was significantly reduced in both neonate and adult isolated vascular samples; however, the expression of tubular markers remained unchanged between groups (Supplementary Figure S1). Similarly, we found that the expression of these and other tubular markers in the microarray was similar amongst the groups (Supplementary Table S3).

**TABLE 4:** Enriched Biological Pathways functional clusters (EBPfc) affected by MatSep.

**Category**	**Term**	**Count**	**%**	***p*-value**	**FE**	**FDR**
**Neonates**						
GOTERM_CC_DIRECT	GO:0005615∼extracellular space	34	17	0.00000	2.49	0.00
GOTERM_BP_DIRECT	GO:0045087∼innate immune response	18	9	0.00000	7.20	0.00
GOTERM_BP_DIRECT	GO:0006954∼inflammatory response	13	6.5	0.00005	4.34	0.07
GOTERM_BP_DIRECT	GO:0043065∼positive regulation of apoptotic process	12	6	0.00091	3.35	1.45
KEGG_PATHWAY	rno04060:Cytokine-cytokine receptor interaction	10	5	0.00065	4.08	0.78
GOTERM_BP_DIRECT	GO:0006955∼immune response	10	5	0.00089	4.00	1.43
GOTERM_BP_DIRECT	GO:0006915∼apoptotic process	10	5	0.01347	2.64	19.59
GOTERM_BP_DIRECT	GO:0043547∼positive regulation of GTPase activity	10	5	0.01722	2.53	24.35
GOTERM_CC_DIRECT	GO:0031012∼extracellular matrix	9	12.2	0.00000	9.92	0.00
GOTERM_BP_DIRECT	GO:0008284∼positive regulation of cell proliferation	7	9.46	0.00940	3.79	12.84
KEGG_PATHWAY	rno04640:Hematopoietic cell lineage	7	3.5	0.00026	7.68	0.31
KEGG_PATHWAY	rno04015:Rap1 signaling pathway	6	8.11	0.00064	7.97	0.74
GOTERM_MF_DIRECT	GO:0005125∼cytokine activity	6	3	0.01663	4.02	20.32
GOTERM_BP_DIRECT	GO:0043123∼positive reg I-kappaB kinase/NF-kappaB	6	3	0.02272	3.71	30.87
GOTERM_BP_DIRECT	GO:0050727∼regulation of inflammatory response	5	2.5	0.00309	8.22	4.85
GOTERM_BP_DIRECT	GO:0030199∼collagen fibril organization	4	5.41	0.00040	27.27	0.58
KEGG_PATHWAY	rno04261:Adrenergic signaling	4	5.41	0.01227	7.87	13.29
GOTERM_BP_DIRECT	GO:0070555∼response to interleukin-1	4	2	0.01056	8.72	15.69
GOTERM_BP_DIRECT	GO:0002250∼adaptive immune response	4	2	0.03103	5.81	39.74
GOTERM_MF_DIRECT	GO:0005518∼collagen binding	3	4.05	0.01396	16.34	15.22
KEGG_PATHWAY	rno04370:VEGF signaling pathway	3	4.05	0.01760	14.05	18.53
GOTERM_BP_DIRECT	GO:0010759∼pos regul of macrophage chemotaxis	3	1.5	0.00548	26.15	8.45
**Adult**						
GOTERM_BP_DIRECT	GO:0070374∼positive regulation of ERK1 and ERK2	6	4.17	0.01355	4.23	19.01
GOTERM_MF_DIRECT	GO:0020037∼heme binding	5	3.47	0.02451	4.51	27.99
GOTERM_MF_DIRECT	GO:0005506∼iron ion binding	5	3.47	0.04604	3.68	46.40
KEGG_PATHWAY	rno04062:Chemokine signaling pathway	5	3.47	0.04885	3.34	51.85
KEGG_PATHWAY	rno04060:Cytokine-cytokine receptor interaction	4	14.3	0.00236	12.75	2.11
GOTERM_BP_DIRECT	GO:0090263∼pos regulation of canonical Wnt path	4	2.78	0.01583	7.51	21.87
GOTERM_BP_DIRECT	GO:0042102∼pos regulation of T cell proliferation	3	10.7	0.00390	30.63	4.84
KEGG_PATHWAY	rno05323:Rheumatoid arthritis	3	10.7	0.00567	23.42	5.01
GOTERM_BP_DIRECT	GO:0007623∼circadian rhythm	3	10.7	0.01247	16.80	14.71
GOTERM_BP_DIRECT	GO:0019221∼cytokine-mediated signaling pathway	3	10.7	0.01487	15.31	17.31
GOTERM_BP_DIRECT	GO:0046329∼negative regulation of JNK cascade	3	2.08	0.01571	15.44	21.72
KEGG_PATHWAY	rno04330:Notch signaling pathway	3	2.08	0.04698	7.61	50.68
GOTERM_BP_DIRECT	GO:0031295∼T cell co-stimulation	2	7.14	0.02834	66.63	30.56

*Data was analyzed using DAVID Functional Annotation Bioinformatics Microarray Analysis. FE = Fisher’s exact test, FDR = Force discovery rate.*

## Discussion

This study shows that MatSep, a rat model of early life stress, dysregulates the expression and activity of several components of the RAS, which normal function is required for an optimal nephron and vascular tissue development during early postnatal life (Guron and Friberg, 2000; Lasaitiene et al., 2003; Walton et al., 2018). Surprisingly, intrarenal AngII levels were reduced in neonates, along with reduced ACE2 expression and activity. These data suggest that other AngII-derived peptides, in addition to the lack of anti-angiogenic effects elicited by Ang 1–7, could be responsible for the exacerbated microvascular density found in MatSep weanlings and adult rats. Furthermore, our transcriptomic analysis indicates that MatSep induces the upregulation of pro-angiogenic and pro-inflammatory gene expression that may contribute to the permanent alterations of the renal microvascular architecture. Our data support the notion that MatSep serves as a strong stimulus during the early postnatal life capable of inducing temporospatial changes in the intrarenal RAS. Overall, these data imply a potential link between postnatal stress and impaired renal structure and function.

Different models of developmental origins of adult disease are based on the exposure to low protein diet, excess of glucocorticoids, induced intrauterine growth restriction and postnatal blockade of the renin-angiotensin system. A common feature of these models is the development of renal damage and hypertension (Zandi-Nejad et al., 2006; Hershkovitz et al., 2007; Ingelfinger and Nuyt, 2012; Stangenberg et al., 2015; Cuffe et al., 2016; Vieira-Rocha et al., 2019; DuPriest et al., 2020; Lamothe et al., 2020). Conversely, MatSep is a chronic behavioral stress model that induces subtle effects on the cardiovascular system in baseline/unstimulated conditions. However, the sensitization of the autonomic, neuroendocrine and immune system becomes a key feature in the enhanced response to secondary stressors (Sanders and Anticevic, 2007; Trombini et al., 2012; Loria et al., 2015). Renal developmental length is species-specific. The initiation of the nephrogenic involves 1/8 of gestation in the humans, 1/3 of gestation in the sheep and 1/2 of gestation in rats. The permanent kidney formation (Metanephros) begins at day 12 in the rat (Seely, 2017). Thus, rats are born during active nephrogenesis. However, once the metanephros stage is achieved, further development and differentiation of tubular and vascular architecture continues throughout P22 (Sequeira Lopez and Gomez, 2004). Importantly, the generation of microvessels in the kidney is not restricted to *in utero* development and can also be activated in response to renal damage later in life (Sequeira Lopez and Gomez, 2004, 2011). During development, renal vascularization occurs in parallel with nephrogenesis, as blood vessels develop through two mechanisms: vasculogenesis (neoformation of vessels) and angiogenesis (sprouting and branching from pre-existent vessels) (Sequeira Lopez and Gomez, 2004, 2011). Newborn renal blood flow (RBF), initially low after birth, progressively increases during postnatal maturation until reaching the adult levels. Newborns display low RBF primarily due to the occurrence of elevated renal vascular resistance (RVR). The increase in RBF observed from postnatal to adult life is caused by progressive reduction of RVR given by the concomitant RAS activation (Nada et al., 2017).

One potential explanation describing the connection between MatSep and an upregulation of RAS components is via the actions of the stress hormones. The chronic behavioral stress associated to MatSep induces the dysregulation of the glucocorticoid (Marais et al., 2008; Chen et al., 2012; Roque et al., 2014), which are well-known for upregulating RAS components, including AGT and angiotensin receptors density and binding in vascular smooth muscle cells and blood vessels (Sato et al., 1994; Schelling et al., 1994; Ullian and Walsh, 1995), adrenal gland (Bobrovskaya et al., 2013), liver epithelial cells (Shelat et al., 1999a) and brain (Aguilera et al., 1995; Shelat et al., 1999b). These actions are mediated by the stimulation of the glucocorticoid response element present in regions of both the AGT and angiotensin receptor promoter (Guo et al., 1995; Matsubara, 1998). AngII synthesis rate depends upon the availability of substrate, AGT, renin, and the activity of the ACE/ACE2 enzymes. Our data shows that MatSep lowered AngII intrarenal concentration while most of the RAS components were upregulated during postnatal life. Thus, we investigated potential explanations and found that ACE in kidneys from MatSep neonates is increased while ACE2 expression and activity are reduced. This data suggests that anti-angiogenic effects mediated by Ang 1–7 could be dramatically attenuated, thus promoting further vessel development (van Esch et al., 2008; Pei et al., 2016; Touyz and Montezano, 2018). Furthermore, it has been shown that AngIII pro-angiogenic effects are mediated by both AT1 and AT2 receptors binding (Kawasaki et al., 1988; Cheng et al., 1994; Del Borgo et al., 2015; Alanazi and Clark, 2019, 2020). Therefore, our data suggests that in the context of glucocorticoid stimulation due to chronic stress, the upregulation of several RAS components could be a major player in the increased renal vascular density via the overstimulation of the AT1 and AT2 receptors along with the reduced Ang 1–7 anti-angiogenic effects. Therefore, factors contributing to AngII degradation in this postnatal milieu, such as increased aminopeptidase A (Enpep) need further investigation.

Kidneys from MatSep weanlings show reduced lectin expression, and both neonates and adult rats show reduced PECAM-1 expression. These data indicate that the alterations in the vascular architecture could be linked to a reduced number of endothelial cells and thus microvascular rarefaction. Potential mechanisms by which MatSep increases microvascular density could be given by the capacity of (pro)renin (Yokota et al., 2008; Uraoka et al., 2009; Zhu et al., 2015) and renin cells (Amaral et al., 2001; Rider et al., 2015) to induce the transcription of angiogenic factors; or linked to the fact that the most of the genes upregulated by MatSep in isolated renal vasculature are associated with angiogenesis during pathophysiological processes. One of them is the microfibrillar-associated protein (Mfap5), which is implicated in the regulation of cell proliferation, differentiation, angiogenesis and apoptosis (Choi et al., 2015; Marti et al., 2015; Saikawa et al., 2018; Boopathy and Hong, 2019). Other gene that shows a strong upregulation in neonates is the fatty acid-binding protein 4 (Fabp4). Fabp4 display both pro-angiogenic (Elmasri et al., 2012; Harjes et al., 2017) and pro-inflammatory (Steen et al., 2017; Dou et al., 2020) actions, therefore its upregulation in the context of chronic stress could be implicated with the enhancement of the vascular density. Finally, despite changes in the angiogenesis regulation directly modulated by MatSep, we are not able to rule out possible vascular remodeling secondary to stress-induced transient blood pressure increases during postnatal life.

It is important to highlight that reduced renal function in MatSep rats at baseline is associated with a moderate increase in proteinuria and no considerable histological renal damage, in rats that otherwise are normotensive and show similar circulating RAS components. Previously, we have reported that adult male MatSep rats display increased sympathetic outflow to the kidneys (Loria et al., 2013a; Loria and Osborn, 2017), showing a reduced number of alpha-adrenergic receptors in isolated renal vasculature. Furthermore, renal filtration capacity was normalized by renal denervation. Taken together, these data suggest that increased microvascular density could also be interrelated with the effects of a greater sympathetic tone on renal hemodynamics. A summary of the current and previous findings related to circulating, vascular and renal RAS in male MatSep rats across the lifespan can be found in Supplementary Table S4.

Nonetheless, this study presents several limitations that may impact the interpretation of the outcomes. First, further studies performing an in-depth characterization of the microvasculature to determine changes in arteriolar alpha SMA actin, ACTA2, MYH11, myosin heavy chain will pinpoint the relationship between increased microvascular density and the vascular wall properties. For instance, it has been shown that postnatal RAS inhibition impairs the development of the microvasculature causing medullary injury. A common characteristic of all these manipulations is the presence of concentric vascular hypertrophy. Thus, determining the effects of MatSep on this type of variables will contribute to the interpretation of the functional consequences of increased renal microvascular density. Second, the isolation of the renal vasculature was performed by the mechanical separation of the vascular from the tubular structures. As such, our samples are enriched in vessels but certainly contains tubular cells. However, we were able to determine that the expression of tubular markers was not different between groups. In addition, this type of procedure is normally associated with a low recovery of the renin cells, which could result in an underestimation of its gene expression. Third, the use of *in situ* hybridization showing whether the increased pro-angiogenic genes are localized on the renal vasculature could address the lack of cell type-specificity. Hence, from all these matters pointed, a single cell analysis will be preferable over the whole vasculature due to the complexity of the sample. The kidney development is based on differential 25-cell type-specific expression of a vast number of genes. Thus, *in situ* hybridization, laser capture microdissection (LCM) and fluorescence-activated cell sorting (FACS) may help to tease the genes specifically involved in the vascular endothelium or vascular wall. Gene expression microarrays provide a powerful tool for studying multifaceted physiological processes. However, implications from microarray data are often impeded by multiple comparisons, small sample sizes, and uncertain relationships to functional endpoints. To date, several genomic studies have been performed in the developing kidney, but fewer have been conducted in the isolated renal vasculature.

Although this study was performed exclusively in male rats, it has been reported that female MatSep rats also display exacerbated AngII-induced hypertension, yet independent of any significant worsening on renal function compared to control littermates (Loria et al., 2013b). The well-described mechanisms by which female rodents show lower blood pressure and protected renal function compared to males is based in the increased pro-vasodilatory factors and greater number of infiltrating T regulatory cells in combination with reduced sympathetic drive, differences that are most likely stimulated by estradiol (Yanes et al., 2008; Garovic and August, 2016). Thus, postnatal stress may exert the sensitization of the renal system via alteration of neuroendocrine, immune and sympathetic responses in a sex-specific manner. Future studies will determine whether intrarenal RAS in female rats respond to MatSep in a similar fashion, while other compensatory factors may account for an optimal renal function during adult life.

In summary, our data show that MatSep is associated with the temporospatial changes in the expression of a cluster of genes, including several RAS components, expressed in the kidney and the renal vasculature. Our results reveal a molecular context to define the critical pathways mediating growth and developmental aberrations resulting in potential changes of function. Although processes such as vasculogenesis and angiogenesis were initially thought to occur only in the developing kidney, now is more accepted that these processes also occur during (physio)pathologic responses during postnatal life. Thus, vasculogenesis and angiogenesis could be activated during the remodeling of the vasculature in response to environmental insults, including this model of psychosocial stress. The kidney is a highly vascularized organ that normally receives ∼20% of the cardiac output. The unique architectural organization of the kidney vasculature with each nephron is critical for the regulation of renal hemodynamics and water and electrolytes balance. However, mechanisms that govern the development of the kidney vasculature are poorly understood. This study provides insights regarding the endowment of renal vessels, with the potential to benefit children and adults with congenital and acquired kidney diseases, vascular diseases, and hypertension.

## Data Availability Statement

The datasets generated for this study are available at https://www.ncbi.nlm.nih.gov/geo/query/acc.cgi?acc=GSE151402.

## Ethics Statement

The animal study was reviewed and approved by Division of Laboratory Animal Resources, IACUC office.

## Author Contributions

CD, AC, GB, KC, and AL designed the experiments. CD, AC, MM, JG, GB, KC, and AL conducted the experiments, analyzed the data, and edited and approved the final version of the manuscript. All authors contributed to the article and approved the submitted version.

## Conflict of Interest

The authors declare that the research was conducted in the absence of any commercial or financial relationships that could be construed as a potential conflict of interest.

## Footnotes

1 http://david.ncifcrf.gov

2 www.graphpad.com

## References

[B1] Aguilera G. Kiss A. Luo X. (1995). Increased expression of type 1 angiotensin II receptors in the hypothalamic paraventricular nucleus following stress and glucocorticoid administration. *J. Neuroendocrinol.* 7 775–783. 10.1111/j.1365-2826.1995.tb00714.x 8563720

[B2] Alanazi A. Z. Clark M. A. (2019). Angiotensin III induces JAK2/STAT3 leading to IL-6 production in rat vascular smooth muscle cells. *Int. J. Mol. Sci.* 20:5551. 10.3390/ijms20225551 31703282 PMC6888423

[B3] Alanazi A. Z. Clark M. A. (2020). Angiotensin III induces p38 Mitogen-activated protein kinase leading to proliferation of vascular smooth muscle cells. *Pharmacol. Rep.* 72 246–253. 10.1007/s43440-019-00035-8 32016850

[B4] Amaral S. L. Roman R. J. Greene A. S. (2001). Renin gene transfer restores angiogenesis and vascular endothelial growth factor expression in Dahl S rats. *Hypertension* 37 386–390. 10.1161/01.hyp.37.2.386 11230305

[B5] Bobrovskaya L. Maniam J. Ong L. K. Dunkley P. R. Morris M. J. (2013). Early life stress and post-weaning high fat diet alter tyrosine hydroxylase regulation and AT1 receptor expression in the adrenal gland in a sex dependent manner. *Neurochem. Res.* 38 826–833. 10.1007/s11064-013-0985-4 23389660

[B6] Boopathy G. T. K. Hong W. (2019). Role of hippo pathway-YAP/TAZ signaling in angiogenesis. *Front. Cell Dev. Biol.* 7:49. 10.3389/fnbeh.2014.00049 31024911 PMC6468149

[B7] Campbell S. Wladimiroff J. W. Dewhurst C. J. (1973). The antenatal measurement of fetal urine production. *J. Obstet. Gynaecol. Br. Commonw.* 80 680–686. 10.1111/j.1471-0528.1973.tb16049.x 4725943

[B8] Centers for Disease Control, and Prevention, (2019). *Chronic Kidney Disease in the United States, 2019.* Atlanta, GA: Centers for Disease Control and Prevention.

[B9] Chen J. Evans A. N. Liu Y. Honda M. Saavedra J. M. Aguilera G. (2012). Maternal deprivation in rats is associated with corticotrophin-releasing hormone (CRH) promoter hypomethylation and enhances CRH transcriptional responses to stress in adulthood. *J. Neuroendocrinol.* 24 1055–1064. 10.1111/j.1365-2826.2012.02306.x 22375940 PMC3380160

[B10] Chen Y. Lasaitiene D. Friberg P. (2004). The renin-angiotensin system in kidney development. *Acta Physiol. Scand.* 181 529–535. 15283767 10.1111/j.1365-201X.2004.01327.x

[B11] Cheng D. Y. DeWitt B. J. McMahon T. J. Kadowitz P. J. (1994). Comparison of pressor responses to angiotensin I, II, and III in pulmonary vascular bed of cats. *Am. J. Physiol.* 266 H2247–H2255. 8023987 10.1152/ajpheart.1994.266.6.H2247

[B12] Choi H. J. Zhang H. Park H. Choi K. S. Lee H. W. Agrawal V. (2015). Yes-associated protein regulates endothelial cell contact-mediated expression of angiopoietin-2. *Nat. Commun.* 6:6943. 10.1038/ncomms7943 25962877

[B13] Cuffe J. S. Burgess D. J. O’Sullivan L. Singh R. R. Moritz K. M. (2016). Maternal corticosterone exposure in the mouse programs sex-specific renal adaptations in the renin-angiotensin-aldosterone system in 6-month offspring. *Physiol. Rep.* 4:e12754. 10.14814/phy2.12754 27122048 PMC4848720

[B14] Dalmasso C. Leachman J. R. Ensor C. M. Yiannikouris F. B. Giani J. F. Cassis L. A. (2019). Female mice exposed to postnatal neglect display angiotensin II-dependent obesity-induced hypertension. *J. Am. Heart Assoc.* 8:e012309. 10.1161/JAHA.119.012309 PMC6912962 31752639

[B15] De Miguel C. Obi I. E. Ho D. H. Loria A. S. Pollock J. S. (2017). Early life stress induces priming of the immune response in kidneys of adult male rats. *Am. J. Physiol. Renal Physiol.* 314 F343–F355. 28971994 10.1152/ajprenal.00590.2016 PMC5899229

[B16] De Miguel C. Obi I. E. Ho D. H. Loria A. S. Pollock J. S. (2018). Early life stress induces immune priming in kidneys of adult male rats. *Am. J. Physiol. Renal Physiol.* 314 F343–F355. 28971994 10.1152/ajprenal.00590.2016 PMC5899229

[B17] Del Borgo M. Wang Y. Bosnyak S. Khan M. Walters P. Spizzo I. (2015). beta-Pro7Ang III is a novel highly selective angiotensin II type 2 receptor (AT2R) agonist, which acts as a vasodepressor agent via the AT2R in conscious spontaneously hypertensive rats. *Clin. Sci.* 129 505–513. 10.1042/cs20150077 26186568

[B18] Dou H. X. Wang T. Su H. X. Gao D. D. Xu Y. C. Li Y. X. (2020). Exogenous FABP4 interferes with differentiation, promotes lipolysis and inflammation in adipocytes. *Endocrine* 67 587–596. 10.1007/s12020-019-02157-8 31845180

[B19] DuPriest E. Hebert J. Morita M. Marek N. Meserve E. E. K. Andeen N. (2020). Fetal renal DNA Methylation and developmental programming of stress-induced hypertension in growth-restricted male mice. *Reprod. Sci.* 27 1110–1120. 10.1007/s43032-019-00121-5 32046425 PMC7539823

[B20] Elmasri H. Ghelfi E. Yu C. W. Traphagen S. Cernadas M. Cao H. (2012). Endothelial cell-fatty acid binding protein 4 promotes angiogenesis: role of stem cell factor/c-kit pathway. *Angiogenesis* 15 457–468. 10.1007/s10456-012-9274-0 22562362 PMC3590918

[B21] Eriguchi M. Lin M. Yamashita M. Zhao T. V. Khan Z. Bernstein E. A. (2018). Renal tubular ACE-mediated tubular injury is the major contributor to microalbuminuria in early diabetic nephropathy. *Am. J. Physiol. Renal Physiol.* 314 F531–F542. 29187372 10.1152/ajprenal.00523.2017 PMC5966765

[B22] Flynn E. R. Lee J. Hutchens Z. M. Jr. Chade A. R. Maric-Bilkan C. (2013). C-peptide preserves the renal microvascular architecture in the streptozotocin-induced diabetic rat. *J. Diabetes Complicat.* 27 538–547. 10.1016/j.jdiacomp.2013.07.002 23994433 PMC3818424

[B23] Franco M. C. Oliveira V. Ponzio B. Rangel M. Palomino Z. Gil F. Z. (2012). Influence of birth weight on the renal development and kidney diseases in adulthood: experimental and clinical evidence. *Int. J. Nephrol.* 2012:608025. 10.1155/2012/608025 PMC3385608 22778952

[B24] Garovic V. D. August P. (2016). Sex differences and renal protection: keeping in touch with your feminine side. *J. Am. Soc. Nephrol.* 27 2921–2924. 10.1681/asn.2016040454 27188841 PMC5042684

[B25] Guo D.-F. Uno S. Ishihata A. Nakamura N. Inagami T. (1995). Identification of a cis-acting glucocorticoid responsive element in the rat angiotensin II Type 1A promoter. *Circ. Res.* 77 249–257. 10.1161/01.res.77.2.249 7614711

[B26] Guron G. Friberg P. (2000). An intact renin-angiotensin system is a prerequisite for normal renal development. *J. Hypertens.* 18 123–137. 10.1097/00004872-200018020-00001 10694179

[B27] Harjes U. Bridges E. Gharpure K. M. Roxanis I. Sheldon H. Miranda F. (2017). Antiangiogenic and tumour inhibitory effects of downregulating tumour endothelial FABP4. *Oncogene* 36 912–921. 10.1038/onc.2016.256 27568980 PMC5318662

[B28] Hershkovitz D. Burbea Z. Skorecki K. Brenner B. M. (2007). Fetal programming of adult kidney disease: cellular and molecular mechanisms. *Clin. J. Am. Soc. Nephrol.* 2 334–342. 10.2215/cjn.03291006 17699433

[B29] Ingelfinger J. R. Nuyt A. M. (2012). Impact of fetal programming, birth weight, and infant feeding on later hypertension. *J. Clin. Hypertens.* 14 365–371. 10.1111/j.1751-7176.2012.00660.x 22672090 PMC8108862

[B30] Johnson A. K. Xue B. (2018). Central nervous system neuroplasticity and the sensitization of hypertension. *Nat. Rev. Nephrol.* 14 750–766. 10.1038/s41581-018-0068-5 30337707 PMC6532772

[B31] Kawasaki H. Takasaki K. Cline W. H. Jr. Su C. (1988). Effect of angiotensin III (des-Asp1-angiotensin II) on the vascular adrenergic neurotransmission in spontaneously hypertensive rats. *Eur. J. Pharmacol.* 147 125–130. 10.1016/0014-2999(88)90641-3 2836216

[B32] Ku E. Lee B. J. Wei J. Weir M. R. (2019). Hypertension in CKD: core curriculum 2019. *Am. J. Kidney Dis.* 74 120–131. 10.1053/j.ajkd.2018.12.044 30898362

[B33] Lamothe J. Khurana S. Tharmalingam S. Williamson C. Byrne C. J. Khaper N. (2020). The role of DNMT and HDACs in the fetal programming of hypertension by glucocorticoids. *Oxid. Med. Cell Longev.* 2020:5751768. 10.1155/2020/5751768 PMC7149440 32318239

[B34] Lasaitiene D. Chen Y. Guron G. Marcussen N. Tarkowski A. Telemo E. (2003). Perturbed medullary tubulogenesis in neonatal rat exposed to renin-angiotensin system inhibition. *Nephrol. Dial. Transplant.* 18 2534–2541. 10.1093/ndt/gfg447 14605275

[B35] Loria A. Reverte V. Salazar F. Saez F. Llinas M. T. Salazar F. J. (2007). Sex and age differences of renal function in rats with reduced ANG II activity during the nephrogenic period. *Am. J. Physiol. Renal Physiol.* 293 F506–F510. 17442728 10.1152/ajprenal.00066.2007

[B36] Loria A. S. Brands M. W. Pollock D. M. Pollock J. S. (2013a). Early life stress sensitizes the renal and systemic sympathetic system in rats. *Am. J. Physiol. Renal Physiol.* 305 F390–F395. 23678041 10.1152/ajprenal.00008.2013 PMC3742864

[B37] Loria A. S. Yamamoto T. Pollock D. M. Pollock J. S. (2013b). Early life stress induces renal dysfunction in adult male rats but not female rats. *Am. J. Physiol. Regul. Integr. Comp. Physiol.* 304 R121–R129. 23174859 10.1152/ajpregu.00364.2012 PMC3543658

[B38] Loria A. S. Kang K. T. Pollock D. M. Pollock J. S. (2011). Early life stress enhances angiotensin II-mediated vasoconstriction by reduced endothelial nitric oxide buffering capacity. *Hypertension* 58 619–626. 10.1161/hypertensionaha.110.168674 21876076 PMC3754790

[B39] Loria A. S. Osborn J. L. (2017). Maternal separation diminishes alpha-adrenergic receptor density and function in renal vasculature from male Wistar-Kyoto rats. *Am. J. Physiol. Renal Physiol.* 313 F47–F54. 28331064 10.1152/ajprenal.00591.2016 PMC5538843

[B40] Loria A. S. Pollock D. M. Pollock J. S. (2010). Early life stress sensitizes rats to angiotensin II-induced hypertension and vascular inflammation in adult life. *Hypertension* 55 494–499. 10.1161/hypertensionaha.109.145391 20026758 PMC2829259

[B41] Loria A. S. Pollock D. M. Pollock J. S. (2015). Angiotensin II is required to induce exaggerated salt sensitivity in Dahl rats exposed to maternal separation. *Physiol. Rep.* 3:e12408. 10.14814/phy2.12408 25999404 PMC4463836

[B42] Marais L. van Rensburg S. J. van Zyl J. M. Stein D. J. Daniels W. M. (2008). Maternal separation of rat pups increases the risk of developing depressive-like behavior after subsequent chronic stress by altering corticosterone and neurotrophin levels in the hippocampus. *Neurosci. Res.* 61 106–112. 10.1016/j.neures.2008.01.011 18329744

[B43] Marti P. Stein C. Blumer T. Abraham Y. Dill M. T. Pikiolek M. (2015). YAP promotes proliferation, chemoresistance, and angiogenesis in human cholangiocarcinoma through TEAD transcription factors. *Hepatology* 62 1497–1510. 10.1002/hep.27992 26173433

[B44] Matsubara H. (1998). Pathophysiological role of angiotensin II Type 2 receptor in cardiovascular and renal diseases. *Circ. Res.* 83 1182–1191. 10.1161/01.res.83.12.1182 9851935

[B45] Mendez M. Gross K. W. Glenn S. T. Garvin J. L. Carretero O. A. (2011). Vesicle-associated membrane protein-2 (VAMP2) mediates cAMP-stimulated renin release in mouse juxtaglomerular cells. *J. Biol. Chem.* 286 28608–28618. 10.1074/jbc.m111.225839 21708949 PMC3151102

[B46] Mitchell K. D. Jacinto S. M. Mullins J. J. (1997). Proximal tubular fluid, kidney, and plasma levels of angiotensin II in hypertensive ren-2 transgenic rats. *Am. J. Physiol.* 273 F246–F253. 9277585 10.1152/ajprenal.1997.273.2.F246

[B47] Murphy M. O. Cohn D. M. Loria A. S. (2017). Developmental origins of cardiovascular disease: Impact of early life stress in humans and rodents. *Neurosci. Biobehav. Rev.* 74 453–465. 10.1016/j.neubiorev.2016.07.018 27450581 PMC5250589

[B48] Nada A. Bonachea E. M. Askenazi D. J. (2017). Acute kidney injury in the fetus and neonate. *Semin. Fetal Neonatal Med.* 22 90–97. 10.1016/j.siny.2016.12.001 28034548 PMC5373985

[B49] Pei N. Wan R. Chen X. Li A. Zhang Y. Li J. (2016). Angiotensin-(1-7) decreases cell growth and angiogenesis of human nasopharyngeal carcinoma xenografts. *Mol. Cancer Ther.* 15 37–47. 10.1158/1535-7163.mct-14-0981 26671566

[B50] Peng H. Carretero O. A. Alfie M. E. Masura J. A. Rhaleb N. E. (2001). Effects of angiotensin-converting enzyme inhibitor and angiotensin type 1 receptor antagonist in deoxycorticosterone acetate-salt hypertensive mice lacking Ren-2 gene. *Hypertension* 37 974–980. 10.1161/01.hyp.37.3.974 11244026

[B51] Ponzio B. F. Carvalho M. H. Fortes Z. B. do Carmo Franco M. (2012). Implications of maternal nutrient restriction in transgenerational programming of hypertension and endothelial dysfunction across F1-F3 offspring. *Life Sci.* 90 571–577. 10.1016/j.lfs.2012.01.017 22365957

[B52] Rider S. A. Mullins L. J. Verdon R. F. MacRae C. A. Mullins J. J. (2015). Renin expression in developing zebrafish is associated with angiogenesis and requires the Notch pathway and endothelium. *Am. J. Physiol. Renal Physiol.* 309 F531–F539. 26202224 10.1152/ajprenal.00247.2015 PMC4572395

[B53] Roque S. Mesquita A. R. Palha J. A. Sousa N. Correia-Neves M. (2014). The behavioral and immunological impact of maternal separation: a matter of timing. *Front. Behav. Neurosci.* 8:192. 10.3389/fnbeh.2014.00192 24904343 PMC4033212

[B54] Saez F. Castells M. T. Zuasti A. Salazar F. Reverte V. Loria A. (2007). Sex differences in the renal changes elicited by angiotensin II blockade during the nephrogenic period. *Hypertension* 49 1429–1435. 10.1161/hypertensionaha.107.087957 17404180

[B55] Saikawa S. Kaji K. Nishimura N. Seki K. Sato S. Nakanishi K. (2018). Angiotensin receptor blockade attenuates cholangiocarcinoma cell growth by inhibiting the oncogenic activity of Yes-associated protein. *Cancer Lett.* 434 120–129. 10.1016/j.canlet.2018.07.021 30031758

[B56] Sanders B. J. Anticevic A. (2007). Maternal separation enhances neuronal activation and cardiovascular responses to acute stress in borderline hypertensive rats. *Behav. Brain Res.* 183 25–30. 10.1016/j.bbr.2007.05.020 17604851 PMC1994156

[B57] Sato A. Suzuki H. Murakami M. Nakazato Y. Iwaita Y. Saruta T. (1994). Glucocorticoid increases angiotensin II type 1 receptor and its gene expression. *Hypertension* 23 25–30. 10.1161/01.hyp.23.1.25 8282327

[B58] Schelling J. R. DeLuca D. J. Konieczkowski M. Marzec R. Sedor J. R. Dubyak G. R. (1994). Glucocorticoid uncoupling of antiogensin II-dependent phospholipase C activation in rat vascular smooth muscle cells. *Kidney Int.* 46 675–682. 10.1038/ki.1994.320 7996788

[B59] Seely J. C. (2017). A brief review of kidney development, maturation, developmental abnormalities, and drug toxicity: juvenile animal relevancy. *J. Toxicol. Pathol.* 30 125–133. 10.1293/tox.2017-0006 28458450 PMC5406591

[B60] Sequeira Lopez M. L. Gomez R. A. (2004). The role of angiotensin II in kidney embryogenesis and kidney abnormalities. *Curr. Opin. Nephrol. Hypertens.* 13 117–122. 10.1097/00041552-200401000-00016 15090868

[B61] Sequeira Lopez M. L. Gomez R. A. (2011). Development of the renal arterioles. *J. Am. Soc. Nephrol.* 22 2156–2165. 10.1681/asn.2011080818 22052047 PMC3250202

[B62] Shelat S. G. Flanagan-Cato L. M. Fluharty S. J. (1999a). Glucocorticoid and mineralocorticoid regulation of angiotensin II type 1 receptor binding and inositol triphosphate formation in WB cells. *J. Endocrinol.* 162 381–391. 10.1677/joe.0.1620381 10467229

[B63] Shelat S. G. King J. L. Flanagan-Cato L. M. Fluharty S. J. (1999b). Mineralocorticoids and glucocorticoids cooperatively increase salt intake and angiotensin II receptor binding in rat brain. *Neuroendocrinology* 69 339–351. 10.1159/000054436 10343175

[B64] Singh R. R. Lankadeva Y. R. Denton K. M. Moritz K. M. (2013). Improvement in renal hemodynamics following combined angiotensin II infusion and AT1R blockade in aged female sheep following fetal unilateral nephrectomy. *PLoS One* 8:e68036 10.1371/journal.pone.068036 PMC3698080 23840884

[B65] Souza L. V. Oliveira V. De Meneck F. Grotti Clemente A. P. Strufaldi M. W. Franco M. D. (2017). Birth weight and its relationship with the cardiac autonomic balance in healthy children. *PLoS One* 12:e0167328. 10.1371/journal.pone.0167328 28095501 PMC5240907

[B66] Stangenberg S. Chen H. Wong M. G. Pollock C. A. Saad S. (2015). Fetal programming of chronic kidney disease: the role of maternal smoking, mitochondrial dysfunction, and epigenetic modfification. *Am. J. Physiol. Renal Physiol.* 308 F1189–F1196. 25656371 10.1152/ajprenal.00638.2014

[B67] Steen K. A. Xu H. Bernlohr D. A. (2017). FABP4/aP2 regulates macrophage redox signaling and inflammasome activation via control of UCP2. *Mol. Cell. Biol.* 37:e0282-16. 10.1128/MCB.00282-16 PMC5214853 27795298

[B68] Su S. Wang X. Pollock J. S. Treiber F. A. Xu X. Snieder H. (2015). Adverse childhood experiences and blood pressure trajectories from childhood to young adulthood: the georgia stress and Heart study. *Circulation* 131 1674–1681. 10.1161/circulationaha.114.013104 25858196 PMC4430378

[B69] Touyz R. M. Montezano A. C. (2018). Angiotensin-(1-7) and vascular function: the clinical context. *Hypertension* 71 68–69. 10.1161/hypertensionaha.117.10406 29203630

[B70] Trombini M. Hulshof H. J. Graiani G. Carnevali L. Meerlo P. Quaini F. (2012). Early maternal separation has mild effects on cardiac autonomic balance and heart structure in adult male rats. *Stress* 15 457–470. 10.3109/10253890.2011.639414 22085295

[B71] Tullos N. A. Stewart N. J. Davidovich R. Chade A. R. (2015). Chronic blockade of endothelin A and B receptors using macitentan in experimental renovascular disease. *Nephrol. Dial. Transplant.* 30 584–593. 10.1093/ndt/gfu361 25438341 PMC4370292

[B72] Ullian M. E. Walsh L. G. (1995). Corticosterone metabolism and effects on angiotensin II receptors in vascular smooth muscle. *Circ. Res.* 77 702–709. 10.1161/01.res.77.4.702 7554116

[B73] Uraoka M. Ikeda K. Nakagawa Y. Koide M. Akakabe Y. Nakano-Kurimoto R. (2009). Prorenin induces ERK activation in endothelial cells to enhance neovascularization independently of the renin-angiotensin system. *Biochem. Biophys. Res. Commun.* 390 1202–1207. 10.1016/j.bbrc.2009.10.121 19879243

[B74] US Renal Data System, (2010). *United States Renal Data System 2010 Annual Data Report: Volume 2: Atlas of End-Stage Renal Disease in the United States.* Washington, DC: US Renal Data System.

[B75] van Esch J. H. Oosterveer C. R. Batenburg W. W. van Veghel R. Jan Danser A. H. (2008). Effects of angiotensin II and its metabolites in the rat coronary vascular bed: is angiotensin III the preferred ligand of the angiotensin AT2 receptor? *Eur. J. Pharmacol.* 588 286–293. 10.1016/j.ejphar.2008.04.042 18511032

[B76] Vieira-Rocha M. S. Rodriguez-Rodriguez P. Sousa J. B. Gonzalez M. C. Arribas S. M. Lopez de Pablo A. L. (2019). Vascular angiotensin AT1 receptor neuromodulation in fetal programming of hypertension. *Vascul. Pharmacol.* 117 27–34. 10.1016/j.vph.2018.10.003 30326265

[B77] Walton S. L. Mazzuca M. Q. Tare M. Parkington H. C. Wlodek M. E. Moritz K. M. (2018). Angiotensin receptor blockade in juvenile male rat offspring: Implications for long-term cardio-renal health. *Pharmacol. Res.* 134 320–331. 10.1016/j.phrs.2018.06.001 29870806

[B78] Xue B. Yin H. Guo F. Beltz T. G. Thunhorst R. L. Johnson A. K. (2017). Maternal gestational hypertension-induced sensitization of angiotensin II hypertension is reversed by renal denervation or angiotensin-converting enzyme inhibition in rat offspring. *Hypertension* 69 669–677. 10.1161/hypertensionaha.116.08597 28223469 PMC5344733

[B79] Yanes L. L. Sartori-Valinotti J. C. Reckelhoff J. F. (2008). Sex steroids and renal disease: lessons from animal studies. *Hypertension* 51 976–981. 10.1161/hypertensionaha.107.105767 18259026

[B80] Yokota H. Takamiya A. Nagaoka T. Hikichi T. Ishida Y. Suzuki F. (2008). Role of prorenin in the pathogenesis of retinal neovascularization. *Hokkaido Igaku Zasshi* 83 159–165. 18546869

[B81] Zandi-Nejad K. Luyckx V. A. Brenner B. M. (2006). Adult hypertension and kidney disease: the role of fetal programming. *Hypertension* 47 502–508. 10.1161/01.hyp.0000198544.09909.1a 16415374

[B82] Zhu T. Miller A. G. Deliyanti D. Berka D. R. Agrotis A. Campbell D. J. (2015). Prorenin stimulates a pro-angiogenic and pro-inflammatory response in retinal endothelial cells and an M1 phenotype in retinal microglia. *Clin. Exp. Pharmacol. Physiol.* 42 537–548. 10.1111/1440-1681.12376 25707593

