# Corrigendum: Intrarenal Renin Angiotensin System Imbalance During Postnatal Life Is Associated With Increased Microvascular Density in the Mature Kidney

**DOI:** 10.3389/fphys.2020.615022

**Published:** 2020-11-16

**Authors:** Carolina Dalmasso, Alejandro R. Chade, Mariela Mendez, Jorge F. Giani, Gregory J. Bix, Kuey C. Chen, Analia S. Loria

**Affiliations:** ^1^Department of Pharmacology and Nutritional Sciences, University of Kentucky, Lexington, KY, United States; ^2^Department of Physiology and Biophysics, Medicine, and Radiology, University of Mississippi Medical Center, Jackson, MS, United States; ^3^Department of Internal Medicine, Hypertension and Vascular Research Division, Henry Ford Hospital, Detroit, MI, United States; ^4^Departments of Biomedical Sciences and Pathology, Cedars-Sinai Medical Center, Los Angeles, CA, United States; ^5^Clinical Neuroscience Research Center, Tulane University, New Orleans, LA, United States

**Keywords:** maternal separation, kidney, renin-angiotensin system, microvascular density, renal transcriptome

In the original article, there was a mistake in Figure 1C as published. **GFR was labeled as mg/day/100 g BW**. The corrected Figure 1C, **where GFR is labeled as ml/min/100 g BW** appears below.

**Figure 1:**
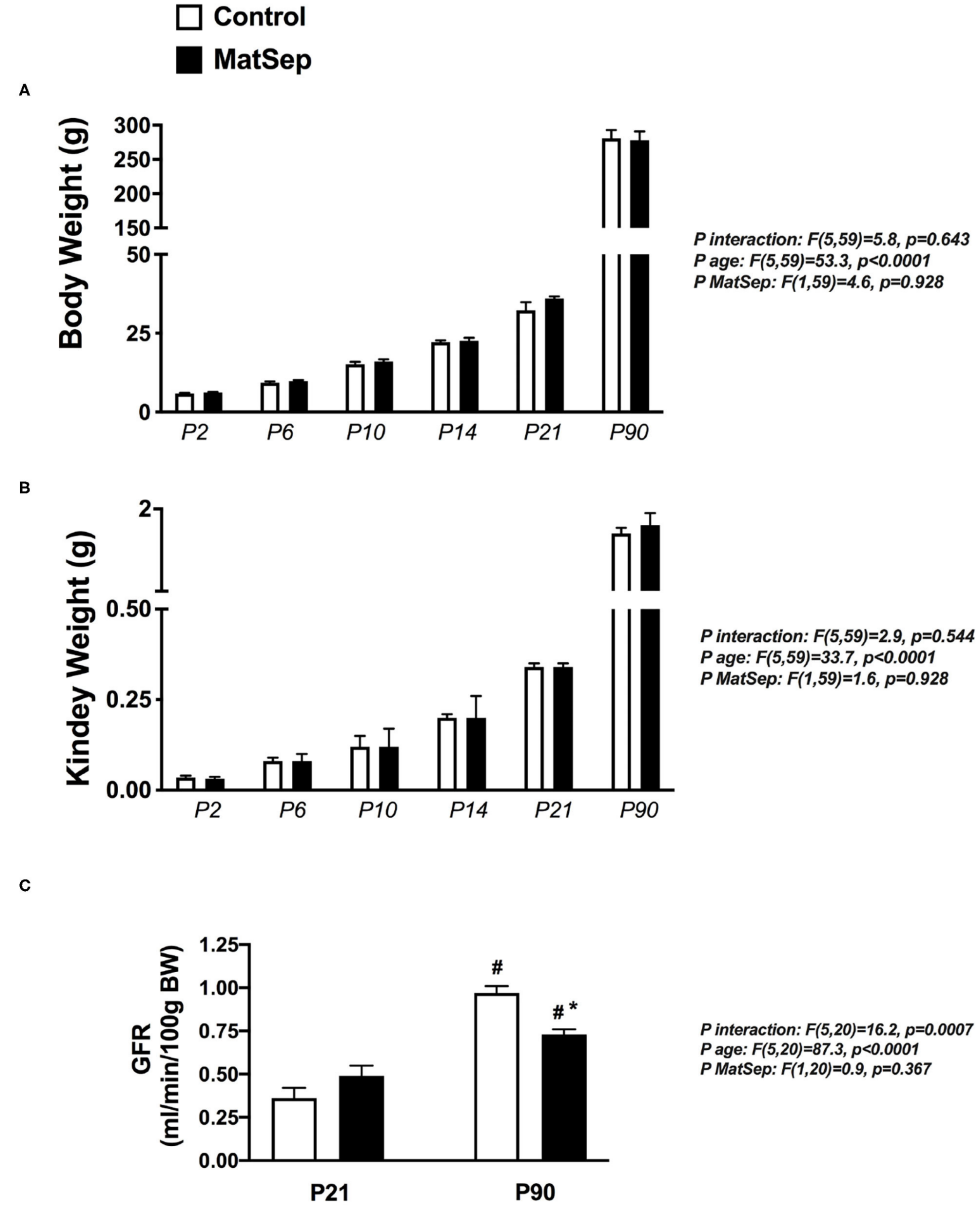
Effect of MatSep on the trajectory from neonatal to adult male rats in: **(A)** body weight, **(B)** Kidney, and **(C)** conscious GFR. ^#^*p* < 0.05 vs. P21, *p < 0.05 vs. C. P: postnatal day. *n* = 6–8 per group.

The authors apologize for this error and state that this does not change the scientific conclusions of the article in any way. The original article has been updated.

