# Supplementary material for: Intrarenal Renin Angiotensin System Imbalance During Postnatal Life Is Associated With Increased Microvascular Density in the Mature Kidney
Source: Front Physiol. 2020 Sep 1;11:1046. doi: 10.3389/fphys.2020.01046 (PMC7491414; doi:10.3389/fphys.2020.01046)
Supplement: Supplementary file 2 [file Table_2.DOCX]

**Intrarenal Renin Angiotensin System Imbalance During Postnatal life is Associated with Increased Microvascular Density in The Mature Kidney**

Carolina Dalmasso^1^, Alejandro R. Chade^2^, Mariela Mendez^3^, Jorge F. Giani^4^, Gregory J. Bix^5^, Kuey C. Chen^1^ and Analia S. Loria^1^

1 Department of Pharmacology and Nutritional Sciences, University of Kentucky

Lexington, KY 40503

2 Department of Physiology and Biophysics; Medicine; and Radiology, University of Mississippi Medical Center, Jackson 39126

3 Hypertension and Vascular Research Division, Department of Internal Medicine, Henry Ford Hospital, Detroit, MI 48202

4 Departments of Biomedical Sciences and Pathology, Cedars-Sinai Medical Center, Los Angeles, CA 90048

5 Clinical Neuroscience Research Center, Tulane University, New Orleans, LA 70112

**Short title: temporospatial effects of early life stress on renal development**

**Corresponding:**

Analia S. Loria, PhD.

Associate Professor

University of Kentucky, Department of Pharmacology and Nutritional Sciences

900 S. Limestone Street, 562 C.T. Wethington Building

Lexington, KY 40536-0200, Phone: 859-218-1414, [analia.loria@uky.edu](mailto:analia.loria@uky.edu)

**Table S1.** Comparison of results from microarray and RT-qPCR. P-value <0.05 is considered statistically significant. Fold change was calculated from control samples. Red= genes >1.2 upregulation, p<.0.05 vs. C; Blue= genes >1.2 downregulation, p<0.05 vs. C; Black= no significant changes vs. C. n= 5 per group.

|  |  | **Results from microarray** | | | | **Results from RT-qPCR** | | | |
| --- | --- | --- | --- | --- | --- | --- | --- | --- | --- |
|  |  | **Neonate**  **MatSep vs. C** | | **Adult**  **MatSep vs. C** | | **Neonate**  **MatSep vs. C** | | **Adult**  **MatSep vs. C** | |
| **Gene symbol** | **Table** | **Fold** | **p-value** | **Fold** | **p-value** | **Fold** | **p-value** | **Fold** | **p-value** |
| ***Mfap5*** | ***4*** | *2.64* | ***0.000*** | *-1.14* | *0.484* | *1.70* | ***0.041*** | *1.35* | *0.096* |
| ***Twist2*** | ***1*** | *1.88* | ***0.001*** | *-1.03* | *0.870* | *3.21* | ***0.000*** | *-0.76* | *0.420* |
| ***Fgl2*** | ***2*** | *1.96* | ***0.000*** | *1.03* | *0.820* | *2.77* | ***0.021*** | *1.25* | *0.285* |
| ***Agtr2*** | ***1*** | *1.58* | ***0.000*** | *1.03* | *0.820* | *1.96* | ***0.047*** | *1.13* | *0.472* |
| ***Tef*** | ***3*** | *1.08* | *0.371* | *1.41* | ***0.000*** | *1.40* | *0.546* | *1.97* | ***0.035*** |
| ***Adam24*** | ***5*** | *1.28* | ***0.002*** | *1.20* | ***0.018*** | *1.49* | *0.053* | *1.33* | ***0.044*** |
| ***Ccl2*** | ***6*** | *1.20* | ***0.007*** | *-1.51* | ***0.000*** | *1.55* | ***0.040*** | *-1.21* | *0.068* |

**Table S2.** Downstream effects of MatSep on gene expression. Fold change was calculated from control samples. Red=genes >1.2 upregulation, p<.0.05 vs. C; Blue= genes >1.2 downregulation, p<0.05 vs. C; Black= no significant changes vs. C. N=5 per group.

|  |  | ***Fold Change*** | |
| --- | --- | --- | --- |
| ***RefSeq*** | ***Gene Symbol*** | ***neonate*** | ***Adult*** |
| ***Early life genes*** |  |  |  |
| NM_001107289 | **Lsm5** | 1.056 | **1.34** |
| NM_001108462 | **Mthfd1l** | 1.051 | **1.28** |
| NM_001165880 | **Egfl8** | 1.035 | **1.20** |
| NM_001191782 | **Nav3** | -1.095 | **-1.43** |
| ***Adult life genes*** |  |  |  |
| ENSRNOT00000028546 | **Dbp** | -1.019 | **1.835** |
| NM_001134593 | **RGD1566380** | 1.009 | **1.770** |
| ENSRNOT00000009975 | **Usp2** | -1.03 | **1.53** |
| ENSRNOT00000026473 | **Cyp1a1** | -1.03 | **1.52** |
| NM_001000103 | **Olr1646** | -1.08 | **1.51** |
| AF347030 | **Epm2a** | 1.060 | **1.502** |
| AF009329 | **Bhlhe41** | 1.127 | **1.493** |
| XM_001069351 | **LOC689056** | -1.13 | **1.45** |
| NM_019194 | **Tef** | 1.077 | **1.416** |
| ENSRNOT00000040695 | **LOC367975** | -1.27 | **1.40** |
| XM_003754807 | **LOC100361671** | -1.09 | **1.36** |
| NM_012651 | **Slc4a1** | -1.178 | **1.331** |
| NM_171992 | **Ccnd1** | -1.03 | **1.26** |
| XR_006358 | **LOC363746** | -1.08 | **1.26** |
| NM_001105871 | **Hrasls** | -1.11 | **1.25** |
| NM_001106304 | **Gprc5b** | -1.03 | **1.24** |
| ENSRNOT00000004785 | **Sytl5** | -1.12 | **1.23** |
| ENSRNOT00000029197 | **Klk14** | -1.16 | **1.22** |
| NM_053523 | **Herpud1** | 1.097 | **1.223** |
| NM_001000646 | **Olr635** | -1.03 | **1.22** |
| NM_001110763 | **Tmem163** | -1.007 | **1.201** |
| XM_233076 | **RGD1562265** | -1.12 | **1.20** |
| NM_001013043 | **Sectm1** | 1.058 | **-1.222** |
| NM_001108781 | **Gmppb** | 1.06 | **-1.22** |
| NM_001106759 | **Exoc3l4** | -1.071 | **-1.226** |
| ENSRNOT00000015976 | **Cd79b** | -1.002 | **-1.232** |
| NR_037346 | **Mir3597-3** | 1.007 | **-1.234** |
| XM_001058423 | **Eda** | 1.14 | **-1.24** |
| ENSRNOT00000022157 | **Chst15** | 1.01 | **-1.27** |
| NM_001257278 | **Il31ra** | 1.178 | **-1.284** |
| NM_001000488 | **Olr1218** | 1.05 | **-1.28** |
| NM_001013894 | **Lilrb4** | -1.083 | **-1.289** |
| ENSRNOT00000021136 | **Rnf125** | 1.21 | **-1.29** |
| NM_001109112 | **Tnfsf13b** | 1.060 | **-1.292** |
| NM_031677 | **Fhl2** | 1.11 | **-1.32** |
| NM_138874 | **Csn1s1** | -1.032 | **-1.321** |
| ENSRNOT00000002089 | **Cd80** | 1.054 | **-1.326** |
| NM_145787 | **Mrgprx3** | 1.028 | **-1.333** |
| NM_001008855 | **RT1-N3** | -1.021 | **-1.347** |
| ENSRNOT00000021397 | **Dpep1** | 1.287 | **-1.423** |
| XM_003753950 | **Klra7** | 1.118 | **-1.449** |
| NM_001001068 | **Olr747** | 1.080 | **-1.449** |
| NM_031530 | **Ccl2** | 1.203 | **-1.511** |
| ENSRNOT00000028626 | **Dtx4** | 1.23 | **-1.53** |
| NR_037614 | **Vof16** | 1.16 | **-1.55** |
| ENSRNOT00000007903 | **LOC680367** | -1.012 | **-1.685** |
| NM_001108214 | **Npas2** | 1.07 | **-1.76** |
| NM_024362 | **Arntl** | 1.11 | **-2.26** |

**Table S3.** Expression of tubular markers in the isolated renal vasculature preparation determined by Affymetric microarray.

**Table S4.** Reports showing RAS status, vascular and renal phenotype in male MatSep, in response to first and second hits across the lifespan.

**
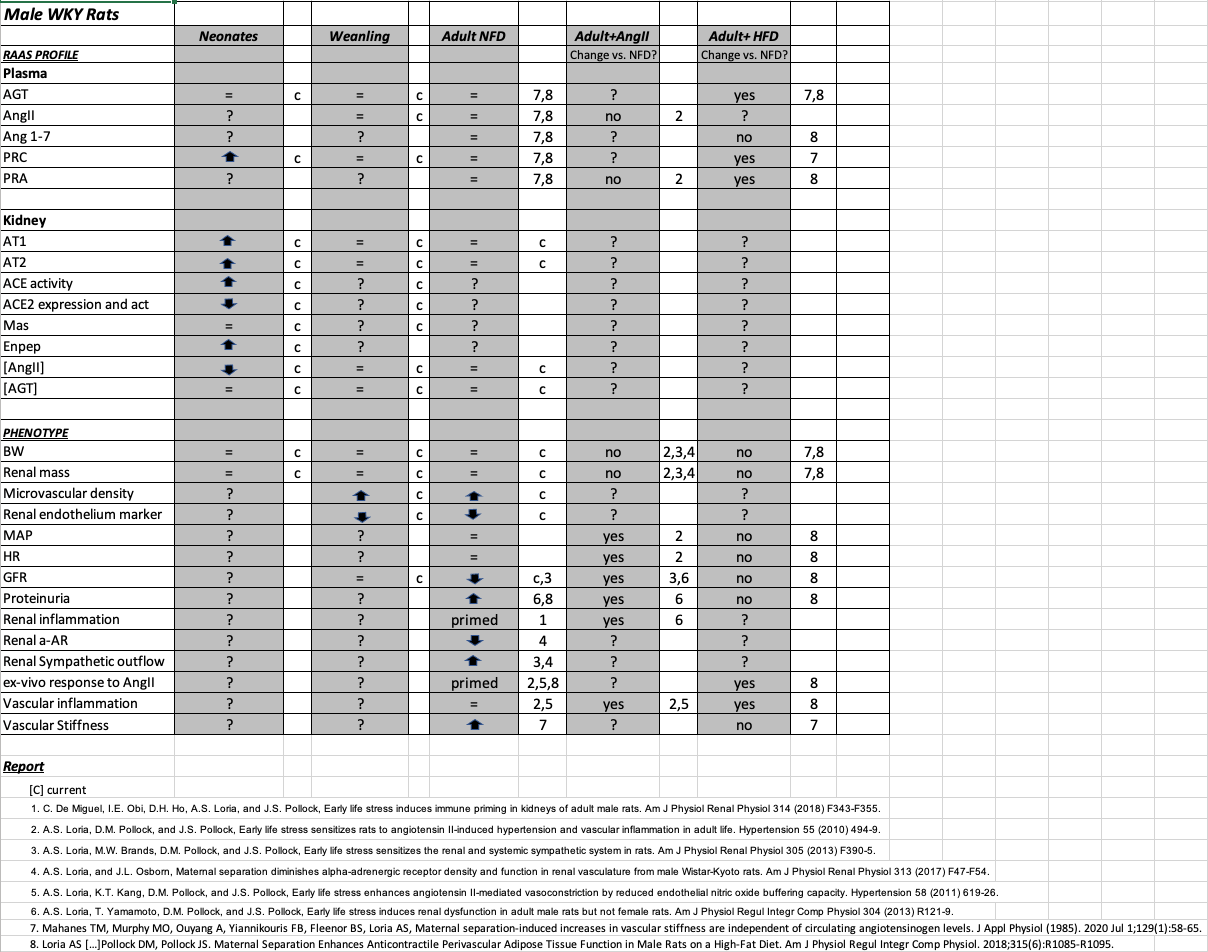
**

**Figure S1.** Expression of PECAM-1 and tubular markers in the isolated renal vasculature preparation determined by RT-qPCR.
